# Anatomical Brain Images Alone Can Accurately Diagnose Chronic Neuropsychiatric Illnesses

**DOI:** 10.1371/journal.pone.0050698

**Published:** 2012-12-07

**Authors:** Ravi Bansal, Lawrence H. Staib, Andrew F. Laine, Xuejun Hao, Dongrong Xu, Jun Liu, Myrna Weissman, Bradley S. Peterson

**Affiliations:** 1 Department of Psychiatry, Columbia College of Physicians & Surgeons and the New York State Psychiatric Institute, New York, New York, United States of America; 2 Department of Biomedical Engineering, Columbia University, New York, New York, United States of America; 3 Departments of Biomedical Engineering, Electrical Engineering and Diagnostic Radiology, Yale University, New Haven, Connecticut, United States of America; University of Maryland, United States of America

## Abstract

**Objective:**

Diagnoses using imaging-based measures alone offer the hope of improving the accuracy of clinical diagnosis, thereby reducing the costs associated with incorrect treatments. Previous attempts to use brain imaging for diagnosis, however, have had only limited success in diagnosing patients who are independent of the samples used to derive the diagnostic algorithms. We aimed to develop a classification algorithm that can accurately diagnose chronic, well-characterized neuropsychiatric illness in single individuals, given the availability of sufficiently precise delineations of brain regions across several neural systems in anatomical MR images of the brain.

**Methods:**

We have developed an automated method to diagnose individuals as having one of various neuropsychiatric illnesses using only anatomical MRI scans. The method employs a semi-supervised learning algorithm that discovers natural groupings of brains based on the spatial patterns of variation in the morphology of the cerebral cortex and other brain regions. We used split-half and leave-one-out cross-validation analyses in large MRI datasets to assess the reproducibility and diagnostic accuracy of those groupings.

**Results:**

In MRI datasets from persons with Attention-Deficit/Hyperactivity Disorder, Schizophrenia, Tourette Syndrome, Bipolar Disorder, or persons at high or low familial risk for Major Depressive Disorder, our method discriminated with high specificity and nearly perfect sensitivity the brains of persons who had one specific neuropsychiatric disorder from the brains of healthy participants and the brains of persons who had a different neuropsychiatric disorder.

**Conclusions:**

Although the classification algorithm presupposes the availability of precisely delineated brain regions, our findings suggest that patterns of morphological variation across brain surfaces, extracted from MRI scans alone, can successfully diagnose the presence of chronic neuropsychiatric disorders. Extensions of these methods are likely to provide biomarkers that will aid in identifying biological subtypes of those disorders, predicting disease course, and individualizing treatments for a wide range of neuropsychiatric illnesses.

## Introduction

Clinicians and researchers have long sought to use brain imaging measures as an aid in clinical diagnosis. In prior attempts to use magnetic resonance imaging (MRI) in the diagnosis of neuropsychiatric illnesses, conventional anatomical measures of putative pathological involvement, such as the overall volume of a brain region or a combination of brain regions, have not proven particularly useful, probably because brain regions that can be identified on MRI scans are anatomically and functionally heterogeneous, which means that for any given brain region, opposing measures of pathological involvement in its various subregions (such as volume loss in one subregion, compensatory hypertrophy in another, and normal volumes in yet another), when combined into an overall volume are dilute and highly variable, producing substantial overlap between diagnostic groups in the distributions of overall volumes. The overlap in distributions, in turn, yields poor sensitivity and specificity when trying to use those measures for clinical diagnosis. [1] However, recent methods in image processing permit measurements of local variation in the morphological features of brain subregions that are thought to be more anatomically and functionally homogeneous than are conventional overall volumes, [1] and that are associated significantly and more uniquely with various neuropsychiatric disorders than are overall volumes of the brain or a brain subregion. [2]–[7]

These measures of local variation in morphological features of the brain have been used to build algorithms that classify the brains of participants according to diagnostic group. For several reasons, however, those diagnostic algorithms have met with limited success when attempting to classify brains that are independent of the imaging dataset used to generate the diagnostic algorithm. First, the morphological measures at each voxel of the brain generally have been assumed to be statistically independent variables when building the classifiers, even though they are not independent of one another. Second, measures at each voxel of the brain generally have been treated as equally informative when building the diagnostic classifiers, when in fact the imaging measures at some voxels are much more diagnostically informative than at other voxels. More important, however, is that diagnostic information is more likely to be represented by the within-subject spatial variation in morphological measures *across* voxels and across brain subregions than by the between-subject variation *within* voxels. For it is the spatial variation across voxels and across brain subregions that is most likely to represent the circuit-based morphological abnormalities that produce and uniquely define a given neuropsychiatric illness. The value of identifying spatial patterns of variation in brain measures across voxels and across brain subregions is much like the value of identifying the spatial patterns for variation in the epidermal ridges of a fingerprint, which define more efficiently and more accurately the identity of an individual than do independent, point-wise measures of displacement of the dermal surface on the tip of a finger. Third, voxel-wise measures of morphological variation yields a vastly larger feature space on which the classification algorithm must operate compared with the feature space for the pattern of spatial variation across voxels, either compromising the validity and reproducibility of the algorithm for a given number of observations (participants) or requiring a much larger number of participants to yield comparable validity and reproducibility of the algorithm.

The fundamental assumption that we make in this paper, and the assumption that constitutes the foundation of our algorithm for diagnostic classification, is that identifying patterns of variation in morphological features that extend over many sets of contiguous voxels and across numerous brain subregions will capture individual disturbances in the morphological features of neural circuits that are unique to each neuropsychiatric disorder being classified. We also believe that rather than using variation at individuals voxels for diagnostic classification, basing the classifications on these patterns of regional variation will reduce the adverse effects of noise on the stability of the diagnostic algorithm, particularly when classifying brains that are independent of the datasets used to generate the algorithm. Herein we demonstrate that a classification using anatomical MR images of the brain if sufficiently precise delineations of brain regions are available across sufficiently distributed neural systems in a sufficiently large number of individuals drawn from healthy and patient populations.

Extant methods for machine-based classification of individual brains in imaging datasets generally [8]–[19] use quantitative imaging data and known clinical diagnoses to learn optimal decision boundaries in the feature space of the imaging dataset that best separate individuals who have differing illnesses. The quantitative features that enter the training dataset (e.g., the Jacobian matrix of the deformation field) have usually been extracted from images using voxel based morphometry [20], [21] (VBM), a technique that provides automated, quantitative, and fine-grained morphological information about the brain. To ensure that the imaging measures are smoothly varying across the brain, the estimated deformation fields are constrained to be locally smooth. These smoothness constraints, however, create difficulties for the imaging measures when trying to classify individuals accurately into diagnostic categories. First, they assume that a voxel in template space (a brain from a healthy individual to which all other brains in the sample are point-wise matched) represents the corresponding anatomical region in the brains across all other individuals in the dataset. This assumption is unlikely to be true, even when the brains of differing individuals have been spatially normalized to the template using high-dimensional deformations, because the smoothness constraints employed when warping a brain into template space, and the variability in anatomy across individual brains, limit the ability of the normalization procedures to match precisely the corresponding anatomical regions across individuals. Second, the smoothness constraint causes deformations in a brain region to be influenced by deformations at all neighboring brain regions, and therefore brain measures derived from these deformation fields do not provide accurate measures of local changes in the brain region of interest. Third, brain measures derived using VBM are defined at each voxel in the brain, and therefore the dimensionality of the feature space is very high, requiring either implementation of techniques that can reduce the dimensionality substantially or else datasets from a much larger number of participants, in order to adequately train and validate the classification algorithms. Together, these limitations of techniques for machine-based classification introduce imprecision when identifying the point-to-point correspondences across brains that are required for accurate classification, as they allow measures from any point in the brain that is warped to a template space to be influenced by the variable features of brain regions at a distance remote from that point. Although more recently developed algorithms for high-order nonlinear warpings [22] significantly reduce these inaccuracies, brain features close to but outside of the regions of interest likely still influence the smoothed deformation fields.

One alternative to VBM, termed “surface morphometry”, may be more accurate in identifying the point-to-point correspondences that are required to define the feature space upon which classification algorithms operate. Surface morphometry first delineates the surface of each brain region precisely and independently from other brain regions, and then it spatially registers independently points on the surface of each brain region to the corresponding points on the surface of the same region in the template brain. One of its major advantages is that point correspondences on brain surfaces are independent of the morphological features of points remote from those surfaces. In addition, its brain measures are defined on fewer voxels compared to VBM-like measures that are defined on all points throughout the entire three-dimensional volume of the brain, thereby dramatically reducing the dimensionality of the feature space on which the classification algorithm is developed and applied. Despite these limitations, the VBM-based algorithms afford the promise of providing completely automated techniques for diagnosing neuropsychiatric disorders, making them potentially attractive for applications within clinical settings. Therefore, comparing the performance of the VBM-based methods with ours and others is essential for future applications of imaging-based diagnostic algorithms. In the future, therefore, we will rigorously compare the performance of our algorithm with that of VBM-based classifications.

## Methods

We present a method to identify valid, naturalistic groupings of the brains based on the spatial patterns of local variation in the morphological features across the surfaces of numerous cortical and subcortical brain regions, with the aim of identifying neural circuit-based disturbances that are unique to specific neuropsychiatric disorders. We capture the patterns of spatial variation in these fine-grained, local morphological features across the surfaces of numerous brain regions in an attempt to represent neural circuit-based patterns of variation in local morphology. The patterns of spatial variations are analyzed using machine-based learning techniques to identify natural groupings of brains. We show that these naturalistic groupings map with high sensitivity and specificity to specific neuropsychiatric disorders. We use computer-generated datasets to validate this general approach, and use human MRI datasets comprising many individuals across a variety of neuropsychiatric disorders to validate and reproduce the specific diagnostic algorithms that classify brains with high sensitivity and specificity as belonging to persons of one specific diagnostic group rather than another.

### Isolating the Brain and Defining ROIs

We isolated the brain from non-brain tissue and defined various brain regions using valid and highly reliable procedures to preclude any rater bias in region definitions. [23]–[25] We first removed inhomogeneities in image intensity [26] and then rotated the brains into a standard head orientation. Next, to ensure the absence of rater bias, we flipped images randomly in the left-right (ear-to-ear) direction prior to region definition and reversed the flips following region definition. We then isolated the brain from non-brain tissue [27] together with manual editing. Therefore, the imaging data were not influenced in any way by knowledge of illness labels prior to generation and testing of the classification algorithm. We defined cortical gray matter using a combination of automated thresholding of gray and white matter and manual editing in orthogonal views. The boundary of each brain region was manually delineated by an expert neuroanatomist using detailed, validated, and well-documented procedures published elsewhere, providing surface delineations that were independent of the influences of other regions [2], [3], [25], [28], [29]. The manual editing needed to isolate the brain from non-brain tissue was 8 hours, to define the cortical mantle was 6 hours, to delineate the amygdala and hippocampus was 4 hours, and to define the basal ganglia was 6 hours of rater time. Therefore, up to 24 hours of a trained, bachelor's level rater was needed to provide initial definitions for all the brain regions used in these classification algorithms. An additional 8 hours was required to check all these definitions in their entirety and to make the necessary revisions by another trained rater before the definition was considered final. Although manual delineations were onerous and time-consuming for a trained expert, they provided valid, precise, and highly reliable region definitions that we believe was important in identifying individual variability in circuit-based morphological characteristics in each participant's brain that was important for the success of our automated classification algorithm.

The various brain regions that we used to train and validate the classification algorithm were defined by more than thirty trained raters over a period of fifteen years. During this 15-year period, several NIMH grants supported the costs for image acquisition and post-processing, with each grant providing support for a five-year period. Trained groups of raters delineated individual brain regions concurrently and while blind to clinical diagnosis and other demographic characteristics for all participants, and region definitions were interleaved randomly across diagnoses over time in our dataset (**Fig. S1**). Therefore, any potential rater bias is unlikely to have confounded the gold-standard clinical diagnoses used to train and validate the automated classification algorithms. Moreover, a senior, more experience rater verified and, when necessary, corrected the definitions before they were made available for further processing, further reducing the likelihood of rater bias. In addition, we reestablished the reliability of each rater every four months in the same set of 10 standardized brains to ensure the absence of drift in delineation of regions over time or raters. When a rater left the laboratory, we trained a new rater who continued to define brain regions on the remaining brains in the dataset. Intraclass correlation coefficients calculated from 10 brains defined by 2 or more raters were greater than (1) 0.91 for the hippocampus [2], (2) 0.89 for the amygdala [2], (3) 0.95 for the caudate and putamen [29], (4) 0.90 for the globus pallidus [29], (5) 0.99 for the cerebral hemispheres [29], (6) 0.95 for the thalamus [30], and (7) 0.98 for cortical thickness [3].

### Quantifying Local Variation in Surface Morphology

We used previously described methods [1]–[3], [28], [31] to quantify precisely the local variations in morphological features across the surfaces of all of the brain regions that we have defined. These methods permit a much finer-grained subdivision of cerebral regions than is possible using more conventional measures of overall volume, thereby providing much greater power to detect localized abnormalities within the region. We first coregistered the cerebrum to a template brain using a similarity transformation that maximized mutual information across the images. [32] We then used a rigid body transformation to coregister individual brain regions to the corresponding template region. Next we warped each brain region nonlinearly to the corresponding template region using a high-dimensional warping algorithm. [33] Each brain region was thus warped to the exact same size and shape as the template region, allowing us to identify points on the surface of each region that corresponded precisely with those of the template region. We then calculated across the entire surface of each region the Euclidean distance of each point from the corresponding point on the template. These distances were positive in sign for protrusions and negative for indentations relative to the template surface. For any given group of participants, this set of signed Euclidean distances constituted a smooth random field on the surface of the template region that quantified local variation in surface morphological features of that region for each participant. A single representative brain was selected as a template rather than an averaged brain because tissue interfaces, such as CSF gray matter or gray-white matter interfaces, are well defined in a single brain. In contrast, in an average brain these interfaces are blurred, thereby increasing registration errors that are subtle but important when distinguishing subtle effects across populations. In addition, precise surface morphometry requires smooth surface devoid of topological defects, which can only be ensured by using a single brain as a template.

We measured the thickness of the cortical mantle in each brain using a 3-dimensional morphological operator to distance-transform the surface of the white matter to the surface of the cortex. [34], [35] This operation calculated cortical thickness as the smallest distance of each point on the external cortical surface from the outermost surface of the white matter. Because these thicknesses were measured in template space, their values inherently accounted for generalized scaling effects in the brain.

### Conformal Mapping of Local Variations in Brain Measures

We used conformal mapping to prepare the surface measures (Euclidean distances and cortical thickness) for spherical wavelet analyses. The purpose of conformal mapping was to transfer the surface measures from the template region onto the surface of a unit sphere, while preserving the angles between vectors on the template and spherical surfaces. Because conformal mapping preserved angles, the shape of a small region on the brain surface was preserved when it was mapped onto the unit sphere. We first used a Marching Cubes algorithm to extract the surface 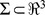 of the template region as a triangular mesh. [36] The extracted surface 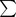 was embedded into the three dimensional space 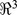 and was assumed to be of genus 0, i.e. the surface was topologically equivalent to a sphere 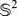 and does not intersect itself. By removing a single point p from the surface 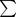 and a point p′ from the sphere 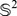, the conformal mapping 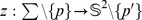 was estimated by solving the partial differential equation
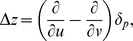
where 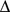 is the Laplace-Beltrami operator, 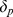 is the Dirac delta function at point p, and u and v are the conformal coordinates in the neighborhood of the point p. This equation was solved using a method for finite element analysis by first mapping the coordinates of the template surface to the coordinates of a plane while preserving the angles between all measures on that surface. [37] Next, a stereographic projection mapped the coordinates of the plane to the coordinates of the sphere. We then used those coordinates to transfer the brain measures from the template surface to the corresponding locations on the sphere's surface.

### Spherical Wavelet Representation

Morphological features are typically measured at more than 10,000 voxels across the surface of a template region. Although a classification algorithm can in principle diagnose an individual in a high-dimensional feature space, an imaging dataset containing many thousands of brains would be required to generate valid classification boundaries that have sufficient reproducibility and generalizability to be potentially useful in a clinical setting. Because even the largest imaging datasets contain images from only a few hundred participants, however, the dimensionality of the feature space must be reduced to generate an algorithm that has valid classification boundaries. Therefore, we elected to use morphological measures defining the two-dimensional manifold of regional surfaces rather than morphological measures for the entire three-dimensional volume of the corresponding regions.

Moreover, our interest was not in classifying individual points on the surface, but instead in classifying the spatial patterns of variation in those measures across the surface of each region. Therefore, before applying clustering (i.e., classification) techniques to the two-dimensional surface dataset, we first captured that spatial pattern in variation of our measures in a manner that also reduced further the dimensionality of the imaging dataset. We accomplished this by applying a spherical wavelet transform to surface measures that had been conformally mapped onto a unit sphere. This wavelet transform generated scaling coefficients that encoded the spatial variation in our measures on the unit sphere at varying degrees of spatial resolution.

Because wavelets have local support – i.e., they equal zero outside a small region – in both the spatial and frequency domains, they have been used to represent functions at multiple levels of spatial detail. A wavelet transform, through dyadic translations and dilations of a mother wavelet and scaling function, generates coefficients for a function such that only a small subset of the coefficients can represent the function with a high degree of precision, thereby permitting compression and efficient processing of imaging data. The wavelet function 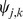 and the scaling 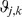, at a resolution 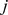 and an index 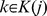 (K(j) is a set of integers at resolution 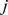 indexing the position of the wavelet and scaling function), is a linear combination of the scaling functions 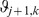 at the higher resolution 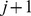. Therefore, the wavelet and scaling functions are self-similar at differing spatial resolutions. If 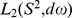 is the space of all scalar functions having finite energy over the sphere 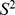, a multi-resolution analysis generates a sequence of closed subspaces 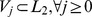, such that: (1) 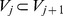, that is 
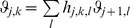
, (2) 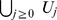 is dense in 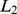, and (3) the set of scaling functions 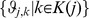 is a Riesz basis of 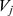. The wavelet functions 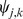 form the bases of the difference space 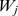 between two successive levels of representation, that is 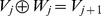. In addition to local support, the wavelet functions have vanishing moments: If wavelets 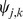 have N vanishing moments, then there exist N independent polynomials 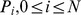 such that 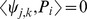, for all 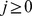,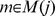, where 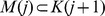 is an index set.

**Figure 1:**
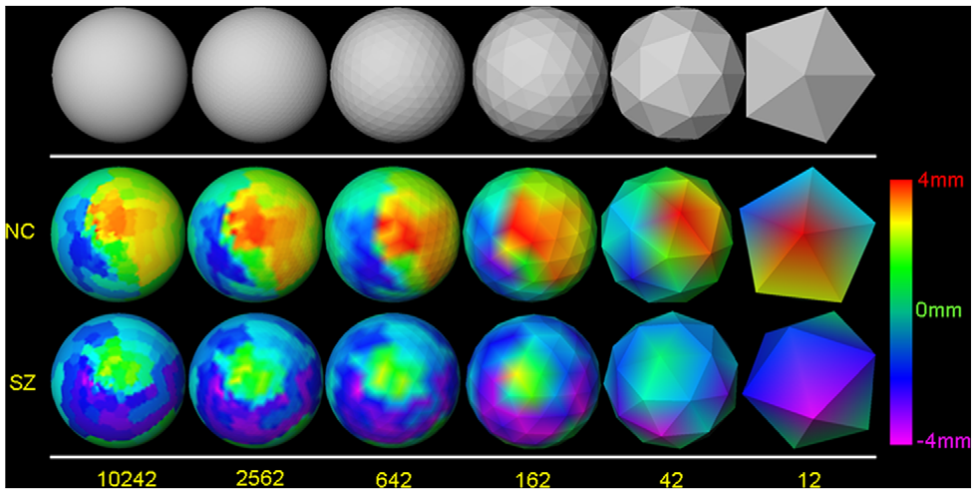
Scaling coefficients at decreasing spatial resolutions. The numbers of vertices in the triangulated mesh at each level of resolution are indicated at the bottom of the figure. The meshes with 12 and 162 vertices correspond to resolutions 0 and 2, respectively, of the spherical wavelet transformation. *Top Row*: Approximating a unit sphere at decreasing spatial resolutions. *Middle and Bottom Row*: Examples of scaling coefficients at decreasing resolutions for local variations in the surface morphological features of the right hippocampus in a representative healthy participant (NC) and a person with Schizophrenia (SZ). The scaling coefficients are color encoded and displayed at the vertices of sphere at the various resolutions. Protrusions with respect to a template surface are encoded in Red and Yellow, and indentations in the surface are encoded in Violet and Blue. Green indicates no difference in the surface. The scaling coefficients were computed by first using conformal mapping to map local variations in surface morphological features onto a unit sphere and then applying the lifted interpolate transformation to the mapped variations. At the lowest resolution, i.e. *Resolution 0* of the wavelet transformation, scaling coefficients at the 12 vertices of the icosahedron very coarsely approximate the high-resolution variations in local morphological features. Using scaling coefficients at low spatial resolutions for classification greatly reduces the dimensionality of the feature space.

Historically, wavelet transforms were initially limited to infinite 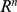 spaces [38], but they subsequently were extended to finite 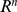 spaces. [39] More recently, they have been extended to analyze scalar functions defined on a sphere using lifting schemes. [40], [41] A lifting scheme builds biorthogonal wavelets that are smoother and that have more vanishing moments than do simple scaling and wavelet functions. A forward analysis of a spherical wavelet transform begins computing coefficients for a function at the highest spatial resolution, continuing until it reaches the lowest possible spatial resolution. At each resolution, unlifted wavelet coefficients are computed, and then these coefficients are lifted to compute the wavelet coefficients, 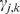, and the scaling coefficients, 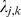. Synthesis, or the inverse transform, in contrast, begins by computing the coefficients at the lowest resolution and ends at the function with the highest resolution. We used two differing methods of wavelet analyses to compute scaling coefficients for our surface measures: (1) the linear lifted method, which used information from the two nearest neighbors 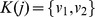 at a location on the mesh of the unit sphere to interpolate the transformation, and (2) the lifted butterfly method, which used information from all eight neighbors 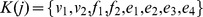 on the mesh of the sphere to compute the scaling coefficients (**Fig. 1**).

### Controlling for Nuisance Variables

Brain measures may differ because of differing ages, gender, or whole brain volume (WBV) across individuals, in addition to differences caused by pathological processes in neuropsychiatric illnesses. To ensure that our method generated naturalistic groupings that represent only the underlying pathological processes and not age and sex effects, we (1) matched our groups on age and sex, and (2) computed brain measures that were corrected for age, gender, and WBV. We had also assessed the effects of higher-order age terms but the linear effects alone were significant, and therefore, we only used linear terms to control for the effects of age. Because brain measures were computed on brains that were normalized into the coordinate space of a template brain of a healthy individual, they were inherently corrected for differing WBV across individuals. These brain measures were further corrected for age and gender effects by applying linear regression with age and gender as the independent variables and brain measure as the dependent variable.

### Machine Learning

We employed a semi-supervised method for machine learning to identify natural groupings of people in the spatial variation of fine-grained, local morphological features across their brains. Machine-based learning and pattern classification seek to construct algorithms that automatically learn decision rules for classification from experimental datasets, and then it applies the learned rules to classify individuals in other datasets. [42] These methods generally belong to either supervised or unsupervised classes of learning. Consider the pairs of data points 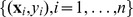, where 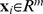 are 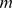-dimensional feature vectors and 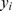 are scalar-valued labels. For our purposes, the vectors 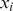 are brain measures and the labels 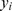 are clinical diagnoses. Supervised learning uses a training sample to learn the mapping between 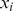 and 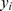 using a parametric or nonparametric function, 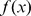. This function encodes a decision rule, or boundary, that optimally separates the feature vectors 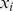 using the labels, 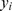. If the labels 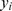 are missing, then methods for unsupervised learning (for example, hierarchical clustering procedures) can be used to discover natural groupings within the data. Our method is semi-supervised because we first applied leave-one-out cross validation to select a set of features that differed significantly between groups of individuals who were already clinically diagnosed, and then we applied hierarchical clustering to the feature vectors to discover naturalistic groupings of individuals in the dataset. We then assessed the validity of these naturalistic groupings by applying leave-one-out and split-half cross-validation procedures to datasets that were independent of those that have been mined to generate the groupings.

#### Discovering Natural Groupings Using Hierarchical Clustering

Hierarchical clustering is a powerful, unsupervised tool for discovering natural structure within a dataset, especially when the groupings of data are unknown a priori. Groupings of brains that are generated from clinical diagnoses alone could frequently misclassify brains if their features do not map tightly to a clinical diagnosis, especially when disturbances affect neural pathways that are common across disorders. To minimize these classification errors, we used hierarchical clustering procedures to discover naturalistic groupings of brain features. We presumed that those common features supported similar computational and behavioral functions, and that the brains that shared them would map more accurately to biologically based groupings of illnesses than would groups of brains classified on the basis of morphological features associated with clinical symptoms as identified by prior classification schemes. A clinical diagnostic label was assigned by a simple majority rule to each of the two naturalistic groupings, so that the known clinical diagnosis affecting the majority of participants in each group provided the diagnostic labels for those groupings. We then used those groupings and labels to classify new brains from a separate dataset. Thus, although the features that generated the classification algorithm were defined by the clinical diagnoses, hierarchical clustering generates groupings based solely on the morphological features and therefore the brains from participants were clustered in groups that shared similar morphological features.

One method for hierarchical clustering generated a sequence of clusters that partitions an imaging dataset from 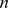 participants, using a measure of dissimilarity between any two groups of feature vectors. Starting with 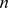 clusters at level 1, with each cluster containing data from exactly one participant, the dataset was partitioned into 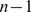 clusters at level 2, 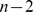 clusters at level 3, and so on, such that only one cluster was present at level 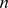. In this sequence of clusterings, any two feature vectors x and x′ were grouped into one cluster at some level, and they remained together at all higher levels. Furthermore, dissimilarity between any two clusters increases with increasing levels, and therefore the groupings of feature vectors that were generated by hierarchical clustering were visualized at various levels using a nonintersecting, binary tree called dendrogram. A dendrogram was drawn to scale to show the dissimilarity between the groups of feature vectors and to help identify natural groupings within the dataset. An unusually large difference in the similarity values across levels may indicate a natural clustering at the lower level. If the similarity values for various levels were evenly distributed across the range of possible values, however, then no particular clustering was more natural than any other.

The use of hierarchical clustering to generate a correct natural grouping of participants depends heavily on the similarity measure used to compare feature vectors, as well as on the method that groups these features using those similarity measures. We defined the similarity between features (sets of scaling factors) as either the Euclidean distance for the feature vectors encoding local variations in the surface morphological measures of a single brain region, or the standardized Euclidean distance for the feature vectors encoding local variations in the surfaces from multiple brain regions. We then used either the average linkage or Ward's linkage on the similarity measures to group the feature vectors. The average linkage calculated the average of the distances between all pairs of feature vectors in the two clusters and then merged as level k the two groups that had the smallest distance between averages. In other words, the average distance 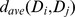 between clusters 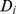 and 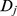 was computed as
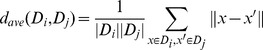
Ward's linkage, in contrast, calculated the distance between two clusters 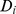 and 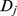 as the increase in the error sum of squares (ESS) of the new cluster 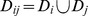, obtained as the union of the individual clusters. For a cluster 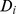, the 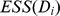 was computed as
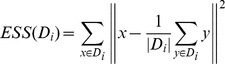
and therefore Ward's distance 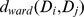 was computed as
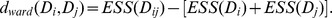
Ward's linkage therefore generated cohesive groupings of participants by minimizing the increase in within-group variance at each level.

Hierarchical clustering as we implemented it may converge to local rather than global optima. Starting with *n* sets of features from *n* brains, hierarchical clustering iteratively combines the features from two sets of brain features to generate a new set of features, thereby reducing the number of sets by one in each step of the iteration. Also at each step, among all possible combinations of two sets, only those two are combined such that the new set optimizes a pre-specified criterion. In our implementation of hierarchical clustering, we used for this pre-specified criterion Ward's distance, which computes within-set variance of brain features. Therefore, in our implementation of hierarchical clustering, two sets of features were combined such that the within-set variance (i.e., Ward's distance) of the new set was smallest among all possible other combinations of two feature sets. This strategy is a greedy one, in that the two sets to be combined at each step are selected from all sets available in that step, and therefore the two groups generated in the last step may not have the least possible within-group variance among all possible two-group combinations of *n* brains. Higher variances in the generated groupings would typically indicate that some brains are assigned to a group erroneously. Such groupings may limit the effectiveness of classification because use of these groupings would possibly create an erroneous assignment of a new participant. Although this is a risk using our implementation of hierarchical clustering, the superb performance and reliability of our classification algorithm suggests that local optima and their attendant errors in classification were very few.

### Independent Validation

We validated the naturalistic groupings using both leave-one-out (LOO) cross-validation analyses and 10 independent split-half replication analyses. In addition, we computed misclassification rates using LOO cross-validation applied to the random halves of data generated in the split-half analyses. In each of the 10 split-half analyses, we (a) partitioned the imaging datasets randomly into two halves, the training set and the test set, (b) generated the classification algorithm using the training set, and then (c) evaluated the performance of the classification algorithm using the second, independent test half of the dataset. Automated, computer-based procedures assigned a brain in the second test dataset to one of the two putative diagnostic groups identified in the training dataset by assessing which of the two groups had morphological features most similar to its own. To assign the brain to one of the two previously defined groups, we first computed the distance between its scaling coefficients and the average coefficients of both groups, and then we assigned the brain to the diagnostic group having the smaller distance from its scaling coefficients. The brain was considered to be misclassified if its diagnosis differed from that of the group label assigned in the training dataset. Assigning each brain in the test dataset one at a time to a putative diagnostic label allowed us to compute misclassification rates in a dataset that was entirely independent of the training dataset that generated the classification algorithm. These split-half procedures were repeated independently for every pair of the 10 training and test datasets, thereby generating 10 independent estimates of the misclassification rates for each pair of clinical diagnoses that we tried to discriminate (e.g. TS vs HC). We computed the means and standard deviations of the misclassification rates across these multiple split-half and LOO cross-validations, which we then used to calculate the sensitivity and specificity of the diagnostic algorithms. It is important to note that, even though the features entering the classification algorithm and the labels assigned to the subsequent naturalistic groupings of brains were determined using previously known clinical diagnoses, it was the hierarchical clustering algorithm alone, operating without information about clinical diagnoses and based solely on the morphological features shared across brains within each dataset, that generated the naturalistic groupings of brains used in the following validation of MRI-based diagnoses.

### Validation using Computer-Generated Datasets

**Figure 2:**
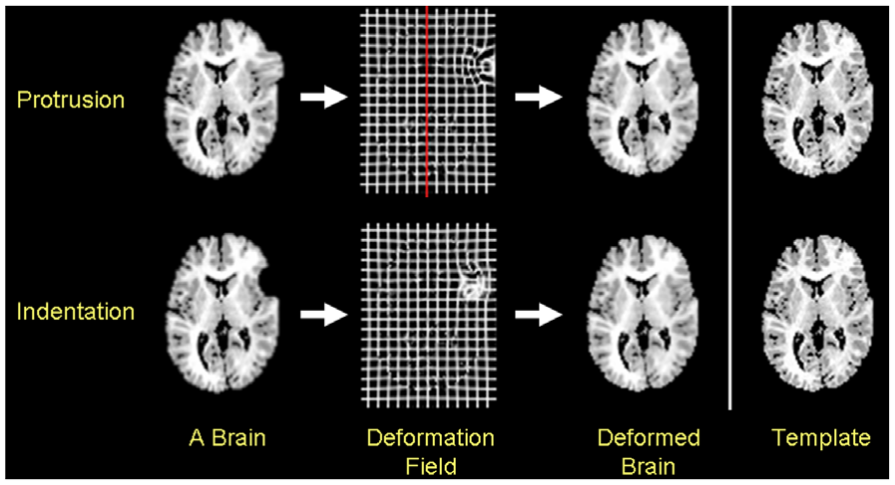
Warping a deformed brain to the template brain. We added deformations to copies of the template brain and then normalized those deformed brains to the undeformed template. The deformed brains were spatially normalized using a method that maximizes mutual information in the gray scale values across the images [32] and then warped the coregistered brain using a method based on fluid dynamics. [33] Because a deformed brain was identical to the template brain except for the added deformation, the deformation field only shows a large spatial deformation in the region of the added deformation.

We generated two synthetic datasets, each with increasing levels of complexity in their surface morphologies, by superimposing spherical indentations or protrusions of known size positioned precisely in the dorsolateral prefrontal cortex (DLPFC) or occipital cortex (OC). [31] These two datasets were used to assess the construct validity of our procedures. The deformation in DLPFC was centered in the midportion of the pars triangularis over the inferior frontal gyrus. The deformation in OC was placed immediately to the left of the interhemispheric fissure on the lowermost portion of the occipital lobe, such that the deformation was tangential both to the cistern immediately superior to the cerebellum and the interhemispheric fissure (**Figs. 2** **&** **3**). We confirmed the accuracy of the placement of these deformations by viewing the deformed brains in the coronal, axial, and sagittal planes. The first dataset was created from copies of a single brain, and the second dataset was created from the brains of 20 different individuals. The second dataset was used to determine the optimal resolution of the scaling coefficients that best discriminate brains of healthy individuals from those with neuropsychiatric illnesses.

**Figure 3:**
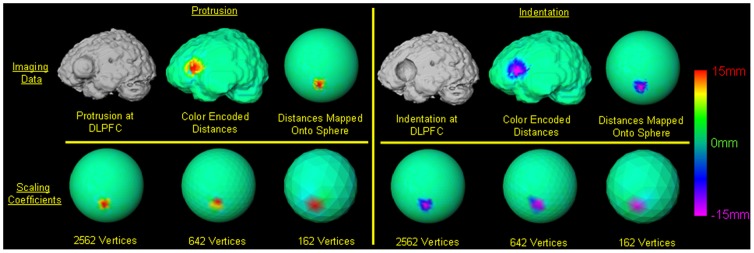
Deformations at the dorsolateral prefrontal cortex (DLPFC) in the template brain. *Top Row*: In copies of the template brain we added a 15 mm wide protrusion or indentation in the DLPFC by centering the deformation over the midportion of the pars triangularis in the inferior frontal gyrus. We placed the deformed brains randomly in the coordinate space of the template brain. The deformed brains were coregistered to the template brain and then we computed the signed Euclidean distances between the surface of the coregistered brain and the surface of the template brain. The signed Euclidean distances were color-encoded and displayed on the template brain, and were mapped onto a unit sphere using a conformal mapping. *Red*: protrusion on the surface; *Violet*: indentations on the surface. *Bottom Row*: The distances on the unit sphere were transformed by applying spherical wavelet transformation to compute scaling coefficients at decreasing resolutions.

#### Synthetic Datasets Constructed from a Single Brain

To create the first set of 10 brains, 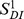, we first generated a deformed brain by adding a 15 mm indentation in the DLPFC in a copy of 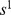, and then placed 10 copies of that brain, each with a differing amounts of translations and rotations, in the coordinate space of the template brain (**Figs. 2** **&** **3**). Similarly, for the second set of 10 brains, 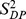, we first generated a deformed brain by adding a 15 mm protrusion to the same location of the DLPFC in a copy of 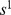, and then we placed 10 copies of that same brain at 10 random locations in the coordinate space. Using identical procedures, we generated a third set 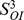 and a fourth set 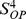 of 10 images by adding an indentation and protrusion, respectively, in the OC region in the copies of 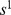 and then placing them at 10 random locations in the space. From these four sets of deformed brains we generated three new sets: (1) 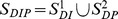 of 20 brains, (2) 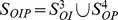 of 20 brains, and (3) 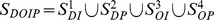 of 40 deformed brains. We then applied our wavelet transform and classification procedures to discover natural groupings in each of these three synthetic datasets. Because brains in these datasets were identical to 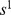 except for the added protrusion and indentation, we expected the brains to be clustered into four groups: one with protrusions in the DLPFC, a second with indentations in the DLPFC, a third with protrusions in the OC, and a fourth group with indentations in the OC.

#### Synthetic Datasets Constructed from Different Individuals

From the brains of 20 healthy individuals, 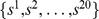, we generated 4 sets of synthetic data: (1) the first set 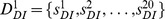 of 20 deformed brains contained a 15 mm indentation in the DLPFC, (2) the second set 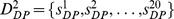 of 20 brains contained a 15 mm protrusion in the DLPFC, (3) the third set 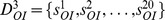 of 20 brains contained a 15 mm indentation in the OC, and (4) the fourth set 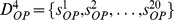 of 20 brains contained a 15 mm protrusion in the OC. From these 4 sets of synthetic data we created 3 new subsets of brains: (1) the first subset 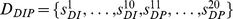 contained 10 brains with indentations and another 10 brains with protrusions in the DLPFC, (2) the second subset 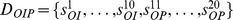 of 10 brains with indentations and another 10 brains with protrusions in the OC, and (3) the third subset 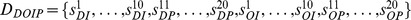 of 10 brains with indentations at DLPFC, another 10 brains with protrusion in the DLPFC, 10 more brains with indentations in the OC, and yet another 10 brains with protrusions in the OC. We then applied the method to automatically discover the natural groupings within these three sets of brains. For each of the two sets 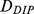 and 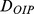, we expected to cluster the brains into two groups: one with only indentations and the other with only protrusions. We expected to cluster brains from the set 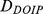 into four groups, one with only protrusions in the DLPFC, another with only indentations in the DLPFC, another with only protrusions in the OC, and a last one with only indentations in the OC.

### Validation using Real-World Data and Gold-Standard Clinical Diagnoses

We validated the natural groupings identified in our classification algorithm using surface morphological measures in a large group of healthy individuals and groups of patients with known clinical diagnoses established by senior clinicians using research-based diagnostic interviews, either the Kiddie-Schedule for Affective Disorders and Schizophrenia (K-SADS) [43] in participants younger than 18 years or the Structured Clinical Interviews for DSM Disorders (SCIDs) [44] in participants older than 18 years. Final clinical diagnoses were assigned following a best-estimate consensus procedure conducted by two board-certified psychiatrists using all available research records but while blind to prior clinical diagnoses. [45], [46] All participants were free of a history of neurological illness, substance dependence, sustained loss of consciousness, and significant medical illness. All participants provided written informed consent for their participation in these studies.

#### Healthy Participants

We acquired imaging data in 42 healthy children (18 males, age 10.5±2.43 years) and 40 healthy adults (22 males, age 32.42±10.7 years). [47] Additional exclusion criteria for healthy participants included a lifetime or a current DSM-IV Axis 1 or 2 disorder, and IQ<80 as measured by the WISC-III [48], WAIS [49], or Kaufmann-Brief Intelligence Test. [50]

#### Tourette Syndrome (TS) Participants

Imaging data was acquired in 71 TS children (59 males, age 11.19±2.2 years) and 36 TS adults (21 males, age 37.34±10.9 years). [47] They were ascertained through the local chapter of the Tourette Syndrome (TS) Association and through the Tic Disorder Clinic of the Yale Child Study Center, New Haven, Conn. Diagnoses were supplemented using the Schedule for Tourette and Other Behavioral Syndromes [51]., and ratings of current and worst ever severity of tic symptoms using the Yale Global Tic Severity Scale. [52]

#### ADHD Participants

We imaged 41 ADHD children (33 males, age 12.6±3.18 years) [28] who were recruited through the general outpatient clinic at the Yale Child Study Center or through advertisements with a local chapter of ChADD (Children with Attention Deficit Disorder). Diagnostic assessments were supplemented using the Conners ADHD Parent and Teacher Rating Scales [53], [54] and the DuPaul-Barkley ADHD rating scale [55]. ADHD subjects with lifetime of Obsessive Compulsive Disorder (OCD), Tourette Syndrome or Tic disorder, and premature birth (gestation ≤36 weeks) were excluded from this group.

#### Bipolar Disorder (BD) I Participants

This group comprised 26 adults with BD (11 males, age 37.65±10.35 years) who were identified from general psychiatry outpatient clinics [56] at the Yale School of Medical Center or the Veterans Affairs Connecticut Healthcare System, or referred by the practitioner. [57] During the history of their illness, all participants met DSM-IV criteria for having had a clearly defined manic episode lasting at least one week.

#### Schizophrenia (SZ) Participants

We acquired imaging data in 65 adults with SZ (41 males, age 42.16±8.71 years). [58] They were identified from general psychiatry outpatient clinics at Yale University Medical Center. All participants had been on their medication for at least 30 days and had not abused substances for at least 60 days. Diagnostic assessments were supplemented using the Positive and Negative Symptom Scale for Schizophrenia. [58], [59]

#### Participants at High or Low Risk for Major Depressive Disorder (MDD)

These individuals belonged to a 3-generation cohort in which the first two generations have been followed for more than 22 years. [3] In the first generation (“G1”), one group of adults was clinically ascertained during treatment of moderate to severe, recurrent, and functionally debilitating MDD; the other group was a sample of matched control adults from the same community who had no discernible lifetime history of depression. The biological offspring of the first generation comprised the second generation (“G2”), and the offspring of the second generation comprised the third generation (“G3”). [3], [60] The participants identified at “high risk” for developing MDD were those members of G2 and G3 who were biological descendants of the MDD group in G1 and those identified at “low risk” were the G2 and G3 biological descendants of the unaffected control group in G1. We acquired imaging data for 131 individuals in G3: 66 (12 children, 54 adults) in the high risk group, and 65 in the low risk group (31 children, 34 adults).

### MRI Pulse Sequence

T1-weighted MR images were acquired on a 1·5 Tesla GE scanner using a sagittal spoiled gradient recall sequence (TR = 24 msec, TE = 5 msec, 45° flip, frequency encoding S/I, no wrap, 256×192 matrix, FOV = 30 cm, 2 excitations, slice thickness = 1.2 mm, 124 contiguous slices). This sequence was selected to provide superior signal-to-noise and contrast-to-noise ratios in high-resolution images having nearly isotropic voxels (1.171×1.171×1.2 mm). For the participants who were at either a high (HR) or a low (LR) familial risk for depression, imaging data were acquired on a 1.5T Siemens Sonata scanner using a 3D MPRAGE sequence with the same parameters [3].

We computed feature vectors from several brain regions, according to the availability of their manual tracings. The feature vector was composed of the scaling coefficients that were derived from the measures of surface morphology. For discriminating brains of individuals with either TS or ADHD from those of healthy comparison individuals and for discriminating TS children from ADHD children, we used feature vectors computed for the morphology of the cortex, globus palladus, putamen, caudate, thalamus, amygdala, and hippocampus. For discriminating brains between persons with other clinical diagnoses, however, we used feature vectors computed from the cortex, amygdala, and hippocampus. The brains of individuals who were at either high or low risk for depression, we used feature vectors computed from the cortical thickness. We applied our classification procedures to discriminate (a) 41 children with ADHD from 42 healthy children, (b) 71 children with TS from 41 children with ADHD, (c) 26 adults with BD from 40 healthy adults, (d) 65 adults with schizophrenia (SZ) from 36 TS adults, (e) 65 adults with SZ from 26 BD adults, (f) 65 adults with SZ from 40 healthy adults, (g) 36 adults with TS from 40 healthy adults, (h) 71 children with TS from 42 healthy children, (i) 65 adults with SZ, 36 adults with TS, and 40 healthy adults, and (j) 65 adults with SZ, 26 adults with BD, and 40 healthy adults.

**Figure 4:**
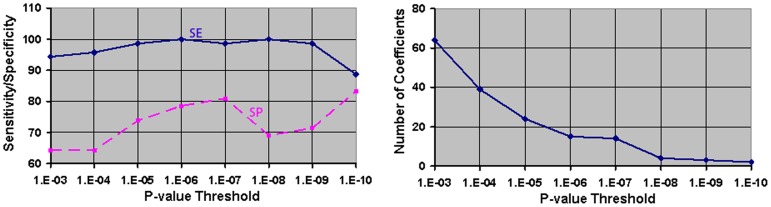
Optimal P-value threshold for scaling coefficients. Naturalistic groupings of the brains were generated using scaling coefficients that differed significantly with at most a specified P-value between groups of participants in our cohort. The optimal P-value of the statistical significance was selected from the plots of sensitivity and specificity, and the number of scaling coefficients, for various P-value thresholds in our cohort of 42 healthy children (HC) and 71 children with Tourette's Syndrome (TS). The scaling coefficients were computed for the right and left amygdalae, hippocampi, global pallidus, putamina, caudate nuclei, thalami, and hemisphere surfaces. At each P-value threshold, we applied hierarchical clustering to all coefficients that differed with at most the specified P-value to generate groupings of the brains. These groupings were analyzed using leave-one-out (LOO) cross validation to compute the sensitivity and specificity of our method for classifying an individual as a healthy child or a child with TS. We independently computed sensitivity and specificities for various P-value thresholds and plotted the sensitivity (SE, solid line) and specificity (SP, dashed line) (*Left*), and the number of coefficients (*Right*), as a function of P-value thresholds. For a P-value threshold<10^−7^, the method classified an individual with both high sensitivity and high specificity. At this P-value threshold, moreover, the number of coefficients was sufficiently reduced, thereby reducing the dimensionality of the feature space. We therefore applied a P-value<10^−7^ as a threshold for classifying an individual among various neuropsychiatric illnesses. **SE**, sensitivity; **SP**, specificity.

We applied our classification scheme to the scaling coefficients that we determined differed at high levels of statistical significance (P-values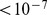) between persons with a specific neuropsychiatric disorder and healthy comparison persons. The p-value for the statistical significance was determined empirically by first applying LOO analysis to scaling coefficients that differed at decreasing p-values and then selecting the p-value associated with the lowest misclassification rates (**Fig. 4**). Using feature vectors that differed at this stringent statistical threshold both reduced the dimensionality of the feature space and identified the features that best discriminated feature vectors for brains in each of the two groups. We then applied hierarchical clustering techniques to the selected scaling coefficients to identify two natural groupings of brains based solely on the similarity of their morphological features.

Each group of brains that hierarchical clustering generated was assigned the diagnosis of the majority of participants contained in that group. Furthermore, although we empirically selected the feature vectors upon which hierarchical clustering was performed, that selection was subsequently validated and their reproducibility was assessed using split-half cross validation. The selected feature vectors therefore should generalize to other similar datasets.

### Identification of Persons at High or Low Familial Risk for Developing a Disorder

We attempted to classify individuals who were at either high familial risk (HR) or low familial risk (LR) for developing Major Depressive Disorder (MDD). The group consisted of 66 HR and 65 LR individuals. Previously we demonstrated the presence of a thinning of the cortical mantle in the lateral aspect of the right cerebral hemisphere and mesial wall of the left hemisphere of the HR compared with LR participants. [3] The cortical thinning was present even in those HR individuals who had never been ill with MDD. Therefore we computed scaling coefficients for local variations in cortical thickness of the HR and LR participants. Classification was more challenging in these high and low risk groups than in groups of already-affected people because most of the HR and LR participants had no lifetime history of MDD, some of the LR participants in generation 3 had parents with prior depression who married into the cohort, and some of the members of the LR group did have a lifetime history of MDD. Therefore, we expected at least some of the participants in these two risk groups to have similar brain features, and we expected misclassification of at least some participants in the HR group as being in the LR group, and vice versa.

## Results

### Synthetic Datasets Constructed from a Single Brain

**Figure 5:**
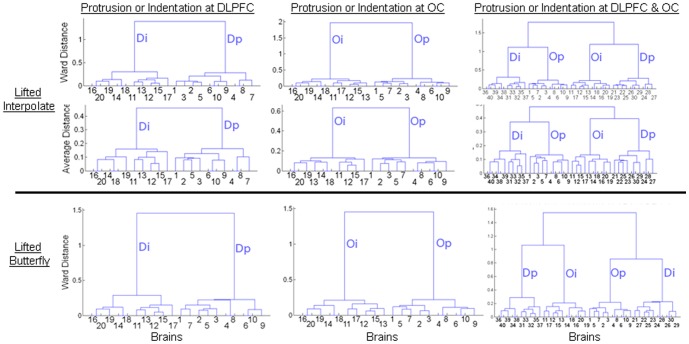
Identifying natural groupings of identical brains containing differing known deformations. *Left Column*: Brains 1 through 10 contained a protrusion, and brains 11 through 20 contained an indentation, in the DLPFC. *Middle Column*: Brains 1 through 10 contained a protrusion, and brains 11 through 20 contained an indentation, in the occipital cortex (OC). *Right Column*: Brains 1 through 10 had a protrusion at the OC, the brains 11 to 20 had an indentation at the OC whereas brains 21 through 30 contained a protrusion at the DLPFC and brains 31 through 40 had an indentation at the DLPFC. The deformed brains were normalized to the template to compute signed Euclidean distances. Those distances were mapped onto a sphere using a conformal mapping. We then applied either the lifted interpolate (*top two rows*) or the lifted butterfly (*bottom row*) to compute the scaling coefficients. Hierarchical clustering computed distances between features using either the average linkage or Ward's linkage. Left and middle dendrograms demonstrate that the brains were correctly clustered into two groups, one with indentations only and the other with protrusions only. Right dendrogram shows that the brains were correctly clustered into four groups: one with only protrusions in the OC, another with only protrusions in the DLPFC, another one with only indentations in the OC, and the last with only indentations in the DLPFC. Furthermore, Ward's distances between groups were larger than the average distances, indicating good separation of groups according to the type of synthetic deformation that was introduced into the data. Ward distances for feature vectors generated using lifted interpolate were generally greater than those generated using lifted butterfly, motivating our subsequent use of the lifted interpolate wavelet to compute scaling coefficients and use of Ward's linkage to cluster brains into naturalistic groups. **Dp**, protrusion in DLPFC; **Di**, indentation in DLPFC; **Op**, protrusion in OC; **Oi**, indentation in OC.

In the dataset with 40 brains with indentations and protrusions in both the DLPFC or OC, our method correctly clustered all brains into 4 groups of 10 brains each, one with only protrusions in the DLPFC, another with only indentations in the DLPFC, a third group with only protrusions in the OC, and a last group with only indentations in the OC (**Fig. 5**). Classification rates were perfect for this synthetic dataset.

Additionally, the same groupings were generated when using either a lifted interpolate or a lifted butterfly wavelet and when using either an Average or a Ward's linkage measure of distance. The analysis using the lifted, interpolate wavelet and the Ward's linkage distance, however, generated more coherent and better separated groups (**Fig. 5**). Thus, in all subsequent analyses we used the lifted interpolate wavelet to compute scaling coefficients and Ward's linkage to generate optimal groupings in hierarchical clustering.

### Synthetic Datasets Constructed from Different Individuals

**Figure 6:**
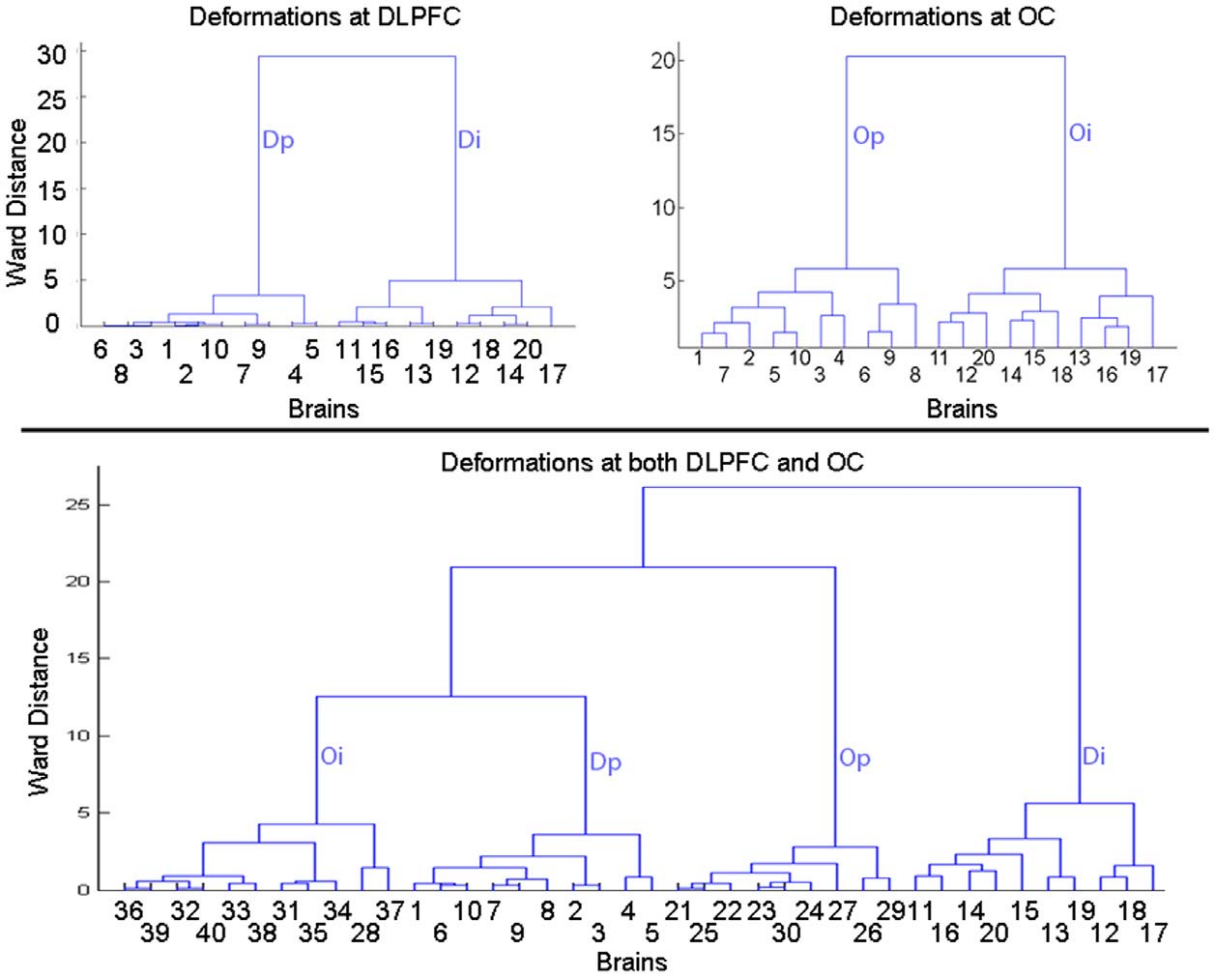
Identifying natural groupings of brains with known deformations from differing individuals. Deformations were placed at either Dorsolateral Prefrontal Cortex (DLPFC) or Occipital Cortex (OC) in brain from 20 individuals. *TopRow*, *Left*: Brains 1 through 10 had protrusions and brains 11 through 20 had indentations at the DLPFC location. *TopRow*, *Right*: Brains 1 through 10 had protrusions and brains 11 through 20 had indentations at the OC location. The brains differed morphologically in addition to the added deformations. Variations in surface morphology from the template brain were analyzed by applying a method for spherical wavelet analysis to compute scaling coefficients at decreasing spatial resolution. The scaling coefficients were grouped by applying hierarchical clustering, which generated one group of brains with indentations only and another group of brains with protrusions only. *TopLeft*: Brains 1 through 10 had protrusions and brains 11 through 20 had indentations at the DLPFC location. Using the scaling coefficients at *Resolution2* that differed significantly between these groups (P-value<10^−7^), the dendrogram shows that the brains were naturally clustered into two groups: one only with indentations, and the other only with protrusions. *Top Right*: Brains 1 through 10 had protrusions and brains 11 through 20 had indentations at the OC location. Using a different scaling coefficients at *Resolution2* that differed significantly, the dendrogram shows that the data were naturally clustered into two groups: one only with indentations, and the other only with protrusions. *Bottom*: Brains 1 through 10 had protrusions, and brains 11 through 20 had indentations, at the DLPFC location. Brains 21 through 30 had protrusions, and brains 31 through 40 had indentations, at the OC location. Using scaling coefficients at *Resolution2*, the dendrogram shows that the data were naturally clustered into 4 groups: one only with indentations at the DLPFC, another only with protrusions at the DLPFC, another only with indentations at the OC, and the last only with protrusions at the OC. However, brain 28 with protrusion at the OC was grouped with brains that had indentations at the OC location. **Dp**; protrusion in DLPFC; **Di**; indentation in DLPFC; **Op**, protrusion in OC; **Oi**, indentation in OC.

Using scaling coefficients at a higher resolution of the wavelet analyses, the method generated groups of brains with indentations in the DLPFC only, protrusions in the DLPFC only, indentations in the OC only, and protrusions in the OC only (**Fig. 6**). At lower resolution, the performance of classification was poor when using datasets constructed from different individuals because differing surface morphology across individuals differentially affected the added deformations. The scaling coefficients at the lower resolution therefore encoded both the inherent variation in surface morphology in these individuals and the added deformations, and therefore the groupings generated by the method were guided by inherent variation in surface morphological features in these individuals. At higher resolutions (i.e. at smaller spatial extents across the cortical surface), the effects of inherent variation in surface morphology was smaller in the scaling coefficients computed across individuals; the scaling coefficients at the higher resolution therefore encoded more accurately the deformation added to the surfaces of these brains. Thus, we used scaling coefficients computed at the higher resolution for classifying individuals subsequently in all of our analyses of real-world datasets.

### Human Datasets

**Figure 7:**
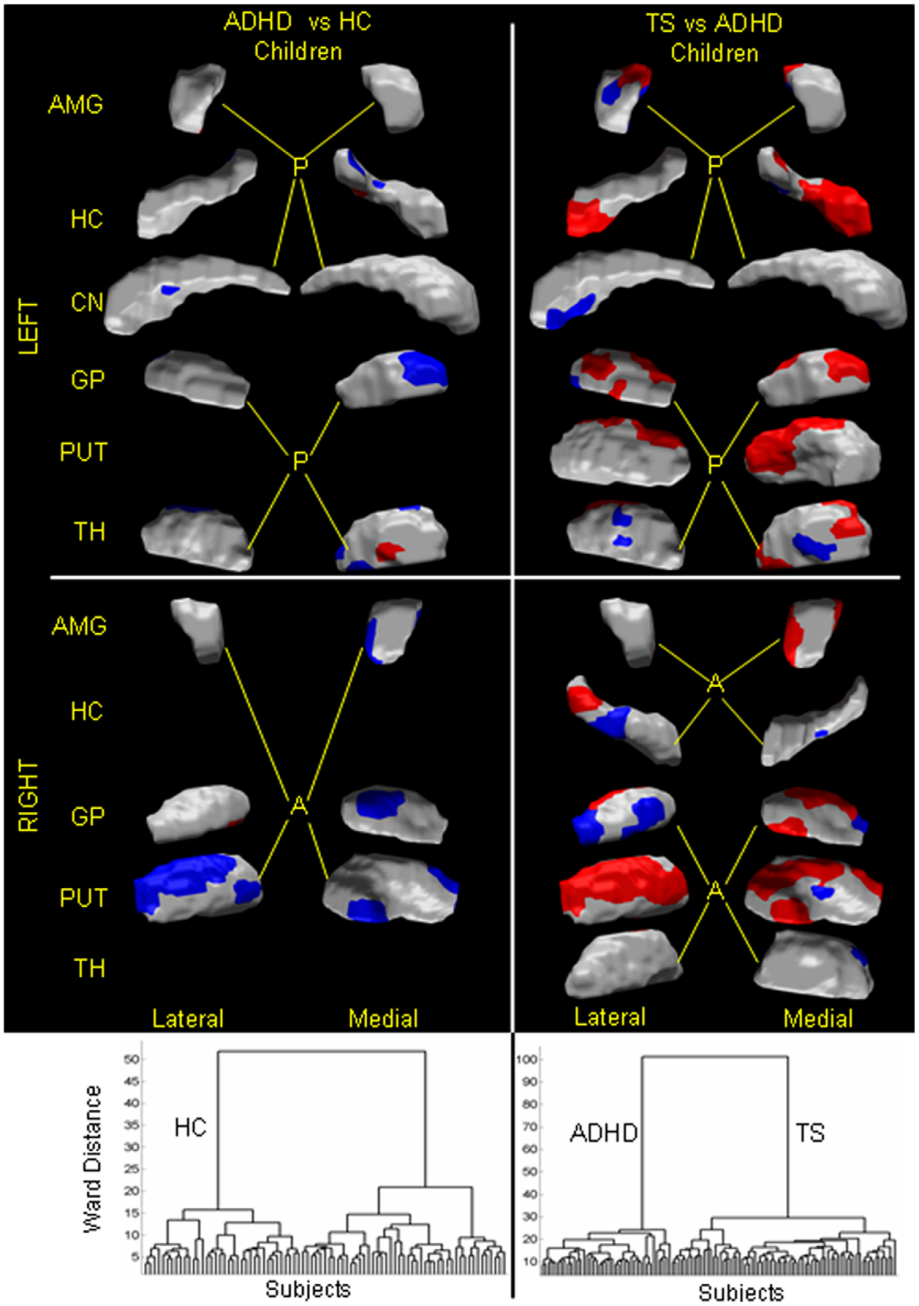
Classifying a child as healthy or with ADHD, or as having either TS or ADHD. In our cohort of 42 healthy children (HC), 41 ADHD children, and 71 children with TS, we first computed scaling coefficients for the left and right globus pallidus, putamina, caudate nuclei, thalami, amygdalae, and hippocampi. We then independently applied hierarchical clustering to those coefficients that differed significantly (P-value<10^−7^) between (1) ADHD children and HC, and (2) TS children and ADHD children. The left dendrogram suggested the presence of two groups: one (labeled as HC) consisted of 36 healthy children, and the other (labeled as ADHD children) consisted of the 41 ADHD children and 6 healthy children. The right dendrogram suggested that the brains were clustered into two distinct groups: one labeled TS only comprised of TS children and the other labeled ADHD only consisted of ADHD children. The adjusted misclassification rates (**Table 1**) were: 11.5% for healthy children and 6.4% for ADHD children; and 0.17% for TS children and 0.5% ADHD children. Therefore, the sensitivity and specificity were: 93.6% and 89.5%, respectively, for classifying a child as an ADHD child; and 99.83% and 99.5%, respectively, for classifying a child as having either ADHD or TS. We plotted the patterns of surface features across the various brain regions that best classified a child. *Left:* The patterns that discriminated ADHD child from healthy child were localized to: lateral and posterior portions of the right putamen; anterior portions of the left and medial portion of the right globus pallidus; ventral portion of the left caudate; posterior and medial portions of the left thalamus; ventral portion of the left amygdala, and anterior and posterior portions of the right amygdala; and posterior portion of the left hippocampus. In *red* are regions with local protrusions, and in *violet* are regions with local indentations in ADHD children compared with the healthy children. *Right:* The pattern of surface features that discriminated between children with TS or ADHD included: anterior, lateral, and dorsal portions of the left globus pallidus, and dorsal, lateral, and medial portions of the right globus pallidus; ventral portion of the left caudate; dorso-medial portions of the left putamen, and lateral, dorsal, and medial portions of the right putamen; dorsal, posterior, and medial portions of the left thalamus, and posterior portion of the right thalamus; dorsal and posterior portions of the left amygdale, and anterior and posterior portions of the right amygdala; anterior and medial portions of the left hippocampus, and lateral portions of the right hippocampus. Regions in *red* are local protrusions, and regions in *violet* are local indentations, in TS children compared with ADHD children. **GP**, globus pallidus, **CN**, caudate nucleus; **PUT**, putamen; **TH**, thalamus; **AMY**, amygdala; **HC**, hippocampus; **A**: Anterior; **P**: Posterior.

**Figure 8:**
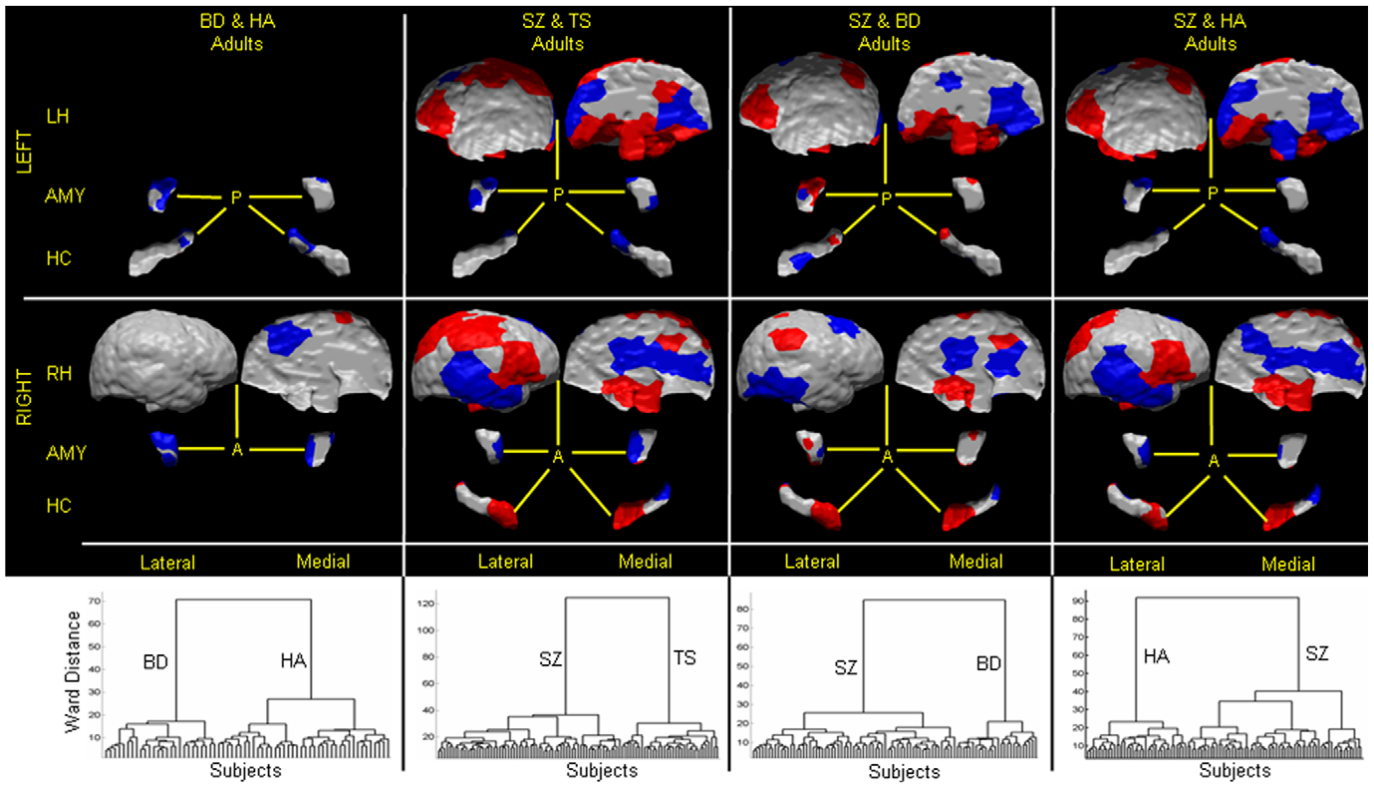
Classifying an adult as healthy or with disorder, or between two neuropsychiatric illnesses. In our cohort of 40 healthy adults (HA), 26 bipolar (BD) adults, 36 TS adults, and 65 adults with schizophrenia (SZ), we first computed scaling coefficients for the left and right hemispheres, amygdalae, and hippocampi. We then independently applied hierarchical clustering to those coefficients that differed significantly (P-value<10^−7^) between (1) BD adults and healthy adults (*1st column*), (2) SZ adults and TS adults (*2nd column*), (3) SZ adults and BD adults (*3rd column*), and (4) SZ adults and HA (*4th column*). The first dendrogram suggested the presence of two groups: one (labeled as HA) consisted of the 40 healthy adults, and the other (labeled as BD adults) consisted of the 26 BD adults. The second dendrogram demonstrated that the brains were clustered into two distinct groups: one containing only TS adults and the other only SZ adults. The third dendrogram also consisted of two distinct groups, one group only of BD adults and the other group only of SZ adults. The fourth dendrogram showed two groups of the brains, one labeled healthy adults consisted of healthy adults only, and the other labeled SZ consisted of all SZ adults and two healthy adults. The adjusted misclassification rates were (1) 3.6% for HA and 0% for BD adults, (2) 0% for both the TS and SZ adults, (3) 0% for both the BD and SZ adults, and (4) 6.9% for SZ adults and 5.5% for healthy adults. Therefore, the sensitivity and specificity were (1) 100% and 96.4%, respectively, for classifying a participant as a BD adult, (2) 100% for classifying an adult as TS or SZ adult, (3) 100% for classifying an adult as BD or SZ adult, and (4) 93.1% and 94.5%, respectively, for classifying a participant as SZ adult. We plotted the patterns of surface features across the various brain regions that best classified an adult. *1st Column* These patterns were localized to: anterior and lateral regions of the left amygdala, and dorsal, lateral, and posterior regions of the right amygdala; posterior regions of the left hippocampus; and dorso-medial regions of the right hemisphere. In *red* are regions with local protrusions, and in *violet* are regions with local indentations in BD adults compared with the healthy adults. *2nd Column* The pattern of surface features (*Bottom*) that discriminated between groups included: anterior and medial portions of the left amygdala, and lateral and posterior regions of the right amygdala; anterior and lateral aspects of the left hippocampus, and posterior portion of the right hippocampus; and dorsolateral prefrontal, parietal, and medial regions of the left hemisphere, and dorsolateral prefrontal, temporal, medial, and parietal regions of the right hemisphere. Regions in *red* are local protrusions, and regions in *violet* are local indentations, in SZ adults compared with TS adults. *3rd Column* The pattern of surface features (*Bottom*) that discriminated between groups included: dorso-medial portions of the left amygdala, and ventro-medial regions of the right amygdala; posterior and lateral aspects of the left hippocampus, and anterior and posterior portion of the right hippocampus; medial dorso-lateral prefrontal, and parietal regions of the left hemisphere, and ventro-posterior, medial, and posterior lateral regions of the right hemisphere. Regions in *red* are local protrusions, and regions in *violet* are local indentations, in SZ adults compared with BD adults. *4th Column* The surface features that best discriminated SZ adults from healthy adults were localized to: dorsolateral prefrontal cortex, superior parietal, and medial regions of the left hemisphere, and temporal, occipital, dorso-lateral, and medial regions of the right hemisphere; dorsal regions of left amygdala, and anterior regions of right amygdala; posterior regions of the left hippocampus, and anterior and posterior regions of the right hippocampus. In *red* are regions with local protrusions, and in *violet* are regions with local indentations in SZ adults compared with the healthy adults. **LH**, left hemisphere; **RH**, right hemisphere; **AMY**, amygdala; **HC**, hippocampus; **A**, anterior; **P**, posterior.

**Figure 9:**
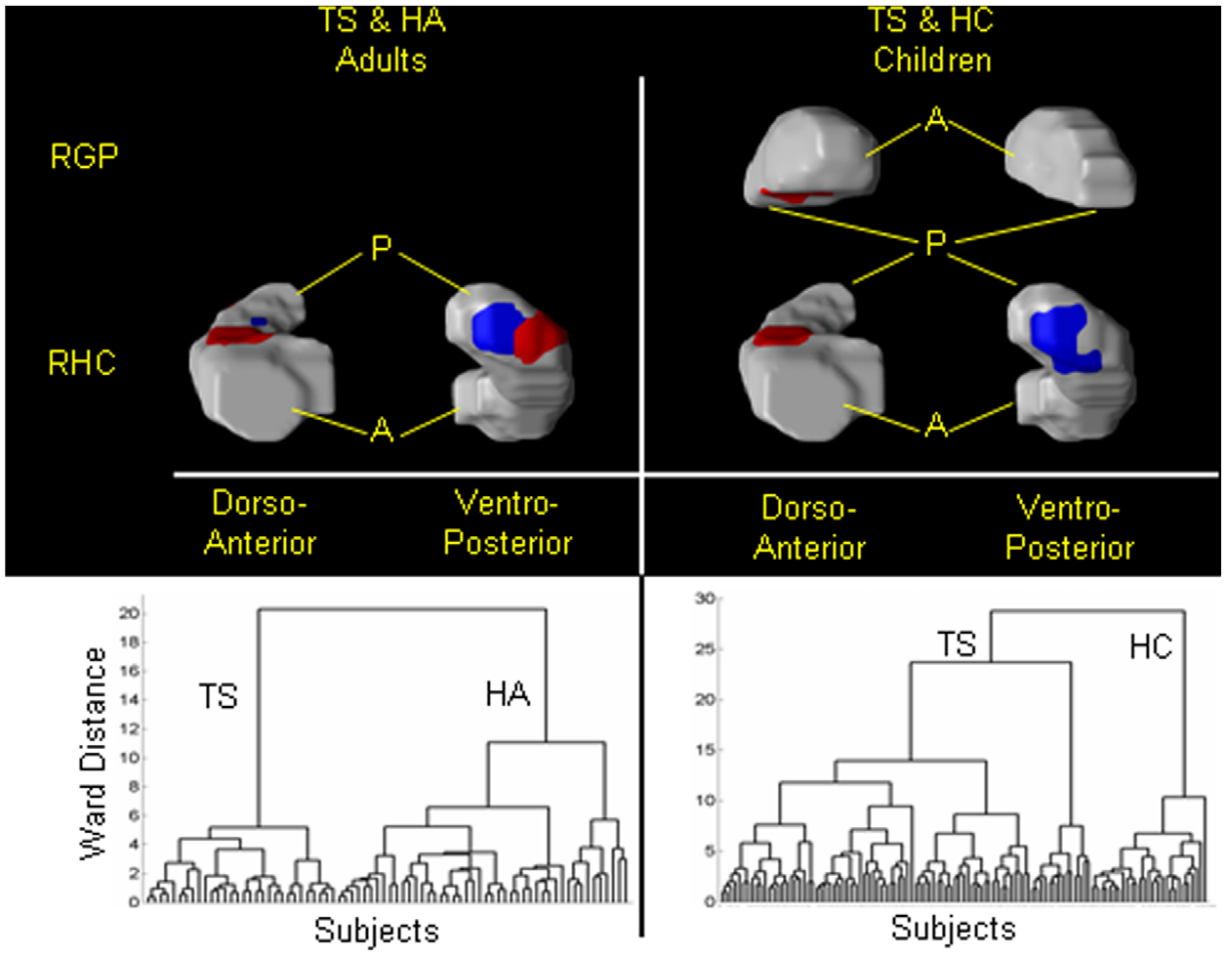
Classifying an individual as a healthy individual or an individual with Tourette Syndrome (TS). In 42 healthy children (HC), 40 healthy adults (HA), 71 children with TS, and 36 adults with TS, we independently applied hierarchical clustering to scaling coefficients that differed significantly (P-value<10^−7^) between (1) adults with TS and healthy adults (*left*), and (2) children with TS and healthy children (*right*). The two largest groups in the right dendrogram were labeled HC (this group consisted of 27 healthy children) or TS children (which consisted of 71 TS and 15 healthy children); and those in the left dendrogram were labeled HA (40 healthy and 6 TS adults) or TS adults (30 TS adults). The adjusted misclassification rates were 5.4% and 21% for the TS children and HC, respectively, and 10% for HA and 16.8% for TS adults. Therefore, the sensitivity and specificity of the method were 94.6% and 79%, respectively, for classifying a child as either healthy child or as having TS, and were 83.2% and 90%, respectively, for classifying a participant as a TS adult. *Left* The dorso-anterior and ventro-posterior regions of the right hippocampus best classified a participant as either a healthy adult or TS adult. Regions in *red* are local protrusions, and regions in *violet* are local indentations, in TS adults compared with healthy adults. *Right* Shown here are the regions in right globus pallidus and right hippocampus where the scaling coefficients differed significantly between TS children and healthy children. The pattern of surface features that discriminated between the two groups included the dorso-anterior portions of the right globus pallidus, and the dorsal and ventro-posterior portion of the right hippocampus. Regions in *red* are local protrusions, and regions in *violet* are local indentations, in TS children compared with healthy children. **RGP**, Right Globus Pallidus; **RHC**, Right Hippocampus; **A**: anterior; **P**: posterior.

**Figure 10:**
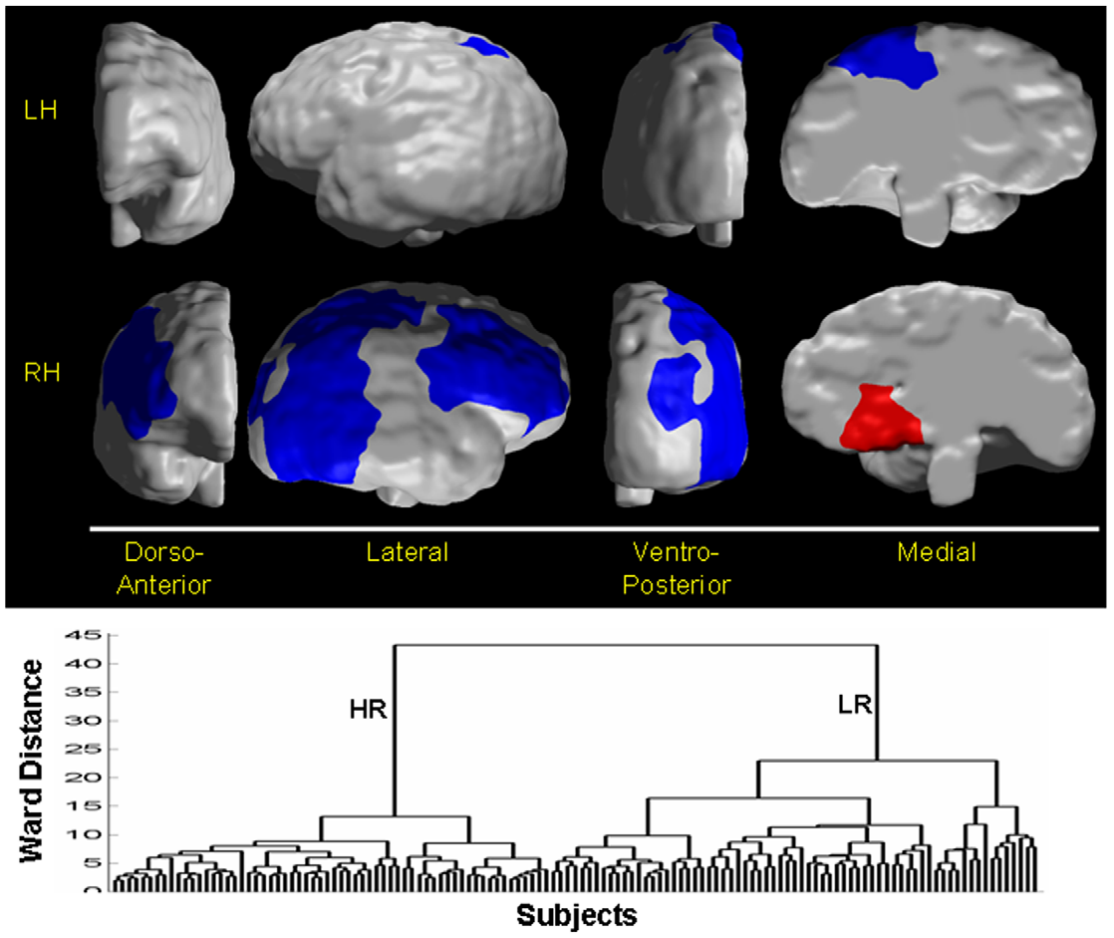
Classifying an individual as high or low familial risk for MDD. In our cohort of 66 High Risk (HR) and 65 Low Risk (LR) participants, we applied hierarchical clustering using Ward's linkages to scaling coefficients computed at *Resolution 2* from the local variations in cortical thickness across the right and the left hemispheres. Each group was assigned a diagnosis using the majority rule, such that a group was labeled HR if the majority of participants in that group belonged to a family with a grandparent who had MDD. Otherwise the group was labeled as LR. Assuming only two groups of participants, the adjusted misclassification rates were 29% for the LR participants and 19% for HR participants. Therefore, the sensitivity and the specificity for classifying an individual as HR were 81% and 71%, respectively. The pattern of cortical thickness that discriminated between the groups included superior regions of the left hemisphere and lateral surface of the right hemisphere. Regions in *red* are local thickening, and regions in *violet* are local thinning, in HR participants as compared with LR participants. The spatial pattern of variation in cortical thickness in the HR compared with the LR group is consistent with the pattern previously identified across risk groups in this sample. [3] **LH**, left hemisphere; **RH**, right hemisphere.

The classification algorithms that our procedures generated were able to discriminate the brains of persons with a specific neuropsychiatric disorder from the brains of healthy persons from the brains of persons who had differing neuropsychiatric disorders, with high sensitivities and specificities (**Table 1**). They discriminated the brains of children with ADHD from HC with 93.6% sensitivity and 88.5% specificity (**Fig. 7** **, left**); children with TS from children with ADHD with 99.83% sensitivity and 99.5% specificity (**Fig. 7** **, right**); adults with BD from HA with 100% sensitivity and 96.4% specificity (**Fig. 8** **, 1st column**); adults with SZ from adults with TS with 99.99% sensitivity and 100% specificity (**Fig. 8** **, 2nd column**); adults with SZ from adults with BD with 99.99% sensitivity and 100% specificity (**Fig. 8** **, 3rd column**); adults with SZ from healthy adults with 93.1% sensitivity and 94.5% specificity (**Fig. 8** **, 4th column**); adults with TS from HA with 83.2% sensitivity and 90% specificity (**Fig. 9** **, left**); children with TS from HC with 94.6% sensitivity and 79% specificity (**Fig. 9** **, right**); and participants at HR for depression from those at LR for depression with 81% sensitivity and 71% specificity (**Fig. 10**). The positive predictive values for each of these classifications were high, ranging from 0.89 to 1.0, except for a value of 0.74 when classifying persons at HR or LR for depression that was not surprising, given we were classifying people at risk for developing an illness and not those with an already-established disorder. Specificities tended to be somewhat lower than sensitivities when classifying patient groups against healthy participants, as misclassifications tended to be more frequent in the healthy participants than when discriminating one neuropsychiatric disorder from another. We suspect that the misclassification of healthy participants may derive from their carrying a brain feature that could place them at greater risk for developing an illness, even though that illness may never become manifest. We have previously demonstrated that kind of brain-based vulnerability in our sample of participants at high or low familial risk for depression. [3] The positive predictive value (PPV), the likelihood that a person diagnosed with an illness using the classification algorithm actually has the illness, was close to 1.0 for most of the datasets. This high PPV could derive in part from be due to the roughly equal number of healthy and affected participants in each classification and therefore could be lower if the algorithms were applied to the brains of persons in the general population, where prevalence rates for neuropsychiatric disorders are typically 2–3%. Nevertheless, certainly 50% or more of persons seeking medical help are likely to have a disorder, and therefore the high PPVs may provide reasonable estimates of the performance of the classification algorithm within a real-world clinical setting.

**Table 1:** Performance of the method using real-world datasets.

Cohort	Demographics	Brain Regions (Number of coefficients)	Misclassification Rates				Sensitivity & Specificity (adjusted)	Positive Predictive Value (adjusted)	Comments & Figure
			LOO (Entire Cohort)	Split Half (10 Training & 10 Test Samples)	LOO Split (20 Split Half Samples)	Adjusted (Entire Cohort)			
**ADHD Children; Healthy Children**	41 participants, 33 males, 12.6±3.18 years; 42 participants, 18 males, 10.5±2.43 years	L&R AMY, L HC, L CN, L&R GP, R PUT, L TH (44)	0.024; 0.0952	0.11±0.11; 0.16±0.10	0.07±0.1; 0.14±0.14	0.064; 0.115	93.6% and 88.5%	0.89	Fig. 1
**TS Children; ADHD Children**	71 participants, 59 males, 11.19±2.2 years; 41 participants, 33 males, 12.6±3.18 years	L&R AMY, L&R HC, L CN, L&R GP, L&R PUT, L&R TH (141)	0.014; 0	0.0105±0.018; 0.005±0.0158	0.0256±0.021; 0±0	0.0017; 0·005	99.83% and 99.5%	0.997	PPV for predicting TS child (Fig. 1)
**SZ Adults; BD Adults**	65 participants, 41 males, 42.16±8.71 years 26 participants, 11 males, 37.66±10.36 years	L&R AMY, L&R HC, LH &RH (93)	0; 0	0.012±0.022; 0±0	0.0108±0.017; 0±0	0.001; 0	99.999% and 100%	1	PPV for predicting SZ adult (Fig. 2)
**BD Adults; Healthy Adults**	26 participants, 11 males, 37.66±10.36 years; 40 participants, 22 males, 32.42±10.7 years	L&R AMY, L HC, RH (82)	0; 0·025	0.044±0.048; 0.043±0.07	0.044±0.07; 0.031±0.033	0; 0.036	100% and 96.4%	0.95	Fig. 2
**SZ Adults; TS Adults**	65 participants, 41 males, 42.16±8.71 years; 36 participants, 21 males, 37·34±10·9 years	L&R AMY, L&R HC, LH & RH (196)	0; 0	0.003±0.009; 0±0	0±0; 0±0	0.003; 0	99.997% and 100%	1	PPV for predicting SZ adult (Fig. 2)
**SZ Adults; Healthy Adults**	65 participants, 41 males, 42.16±8.71 years; 40 participants, 22 males, 32.42±10.7 years	L&R AMY, L&R HC, LH &RH (119)	0.015; 0.025	0.106±0.047; 0.04±0.0459	0.0525±0.045; 0.01±0.02108	0.069; 0.055	93.1%; 94.5%	0.963	Fig. 2
**TS Adults; Healthy Adults**	36 participants, 21 males, 37.34±10.9 years; 40 participants, 22 males, 32.42±10.7 years	R HC (6)	0.11; 0.025	0.15±0.077; 0.18±0.193	0.09±0.09; 0.10±0.1	0·168; 0·10	83.2% and 90%	0.91	Fig. 3
**TS Children Healthy Children**	71 participants, 59 males, 11.19±2.2 years 42 participants, 18 males, 10.5±2.43 years	R HC, R GP (14)	0.014 0.19	0.13±0.177 0.29±0.17	0.09±0.15 0.31±0.15	0.054 0.21	94.6% and 79%	0.90	Fig. 3
**High Risk; Low Risk**	66 participants, 31 males, 33.30±12.90 years; 65 participants, 30 males, 24.79±13.14 years	L&R Cortical Thickness (26)	0.181; 0.246	0.15±0.11; 0.45±0.145	0.142±0.11; 0.4±0.143	0.19; 0.29	81% and 71%	0.74	Fig. 4
**SZ Adults; TS Adults; Healthy Adults**	65 participants, 41 males, 42.16±8.71 years; 36 participants, 21 males, 37.34±10.9 years; 40 participants, 22 males, 32.42±10.7 years	L&R AMY, L&R HC, LH & RH (253)	0.0153;0.22; 0.625	0.08±0.14; 0.533±0.28; 0.49±0.31	0.028±0.045; 0.64±0.31; 0.035±0.27	0.067; 0.11; 0.737			Classifying an individual among 3 groups: (1) SZ adult, (2) TS adult, or (3) healthy adult (Fig. S7)
			0.0153; 0	0.015±0.036; 0.011±0.019	0.006±0.01; 0.01±0.019	0.0245; 0.001	97.76% and 99.999%	0.999	Classifying an individual among two groups: (1) SZ adult, and (2) healthy or TS adult.
**SZ Adults; BD Adults; Healthy Adults**	65 participants, 41 males, 42.16±8.71 years; 26 participants, 11 males, 37.66±10.36 years; 40 participants, 22 males, 32.42±10.7 years	L&R AMY, L&R HC, LH & RH (112)	0.015; 0.11; 0	0.73±0.177; 0.28±0.24; 0.0515±0.057	0.078±0.10; 1±0; 0.097±0.133	0.80; 0.83; 0.046			Classifying an individual among 3 groups: (1) SZ adult, (2) BD adult, or (3) healthy adult (Fig. S7)
			0.14; 0	0.167±0.079; 0.0121±0.022	0.165±0.092; 0.011±0.04	0.14; 0	86% and 100%	1	Classifying an individual among two groups: (1) SZ adult or BD adult, and (2) healthy adult.

We applied our method for discriminating the brains of persons with a specific neuropsychiatric disorder from those of healthy persons or persons with other neuropsychiatric disorders. The misclassification rates were computed by applying (1) leave-one-out (LOO) analysis to the entire cohort of participants, (2) split-half analysis to10 pairs of training and test samples, each sample with half the total number of brains in the entire cohort, and (3) LOO analysis to each of the 10 test and 10 training samples used for the split half analyses. The differences in the misclassification rates computed in (2) and (3) were added to the misclassification rates computed for the entire cohort to compute the adjusted misclassification rates. These adjusted rates were then used to calculate the sensitivity and specificity of our method for discriminating brains. In addition, we computed the Positive Predictive Value (PPV) that measures the proportion of individuals having the illness (true positives) among all individuals classified by the method as having that illness (true positives+false positives). The PPV gives the likelihood that a person diagnosed with an illness using the classification algorithm actually has the illness and was close to 1 in most of the datasets. Because procedures classified individuals with high sensitivity and specificity, multiple and independent split-half analyses provided strong evidence that the procedures were robust to variations in the sampling of participants who generated the classification algorithm. The performance of three-way classifications was poor, however, suggesting an iterative approach with nested two-way classifications for the accurate discrimination of the brains among three clinical conditions. Using the iterative approach for classifying an adult as healthy, with BD, or with SZ, the misclassification rates were 0 for healthy adult, 0·14 for BD adult, and 0·14 for SZ adult. Similarly, for the iterative approach for classifying an adult as healthy, with TS, or with SZ, the misclassification rates were 0.10 for healthy adult, 0.168 for TS adult, and 0.0245 for SZ adult.

**Brain Regions:** these were the regions for which scaling coefficients differed significantly between diagnostic groups at a p-value<10^−7^ and that subsequently were submitted for hierarchical clustering. This column also lists the total number of coeffcients from all brain regions used in each classification.

**ADHD**: Attention Deficit/Hyperactivity Disorder; **HC**: Healthy children; **TS**: Tourette Syndrome; **SZ**: Schizophrenia; **BD**: bipolar disorder; **HA**: healthy adults; **L**: left; **R**: right; **LH**: Left Hemisphere; **RL**, Right Hemisphere; **AMY**: Amygdala; **HC**: Hippocampus; **GP**: Globus Pallidus; **PUT**: Putamen; **TH**: Thalamus; **CN**, Caudate Nucleus; **PPV**: positive predictive value.

### Three-Way Classifications Using Real-World Data and Gold-Standard Clinical Diagnoses

**Figure 11:**
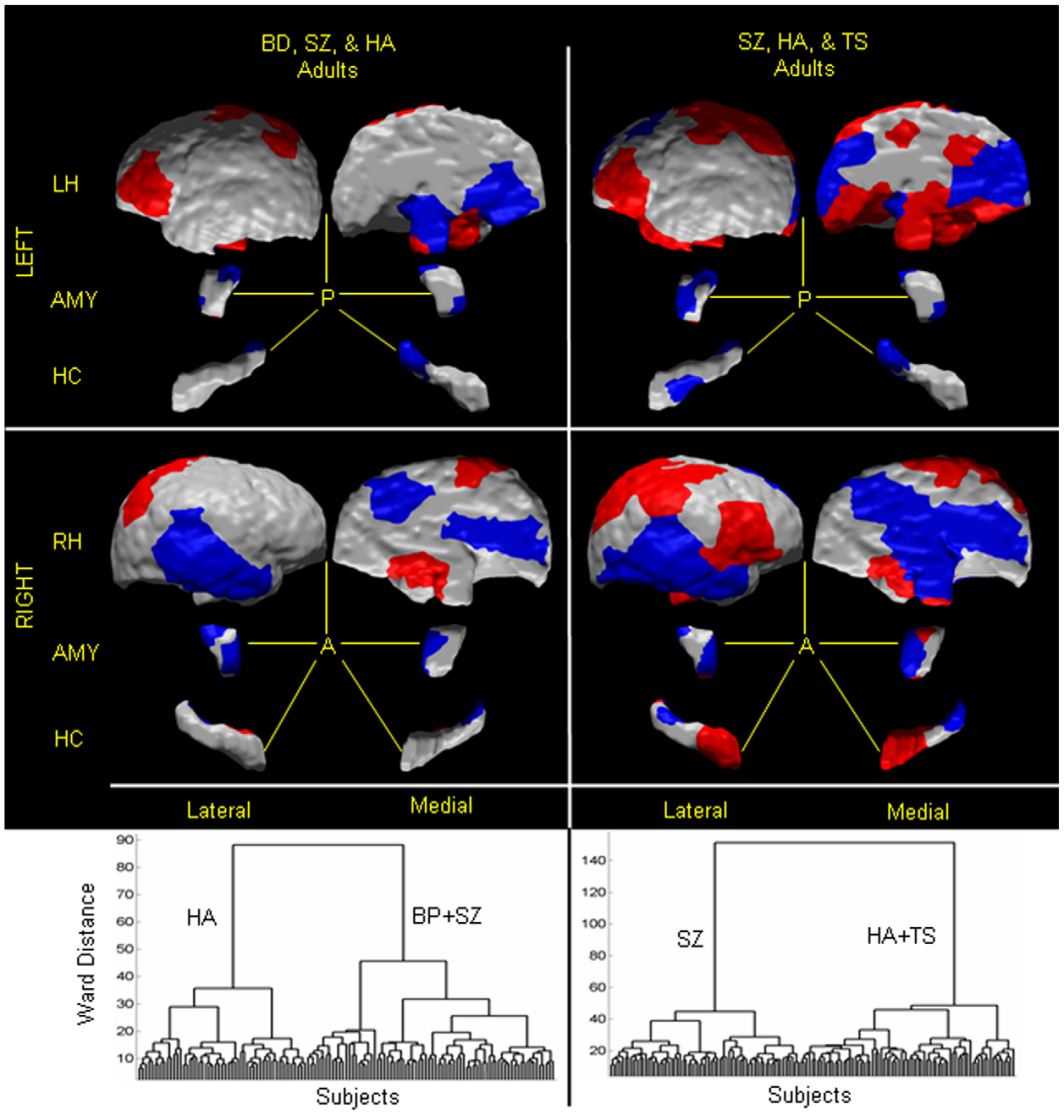
Three-way classifications of an adult. In our cohort of 40 healthy adults (HA), 26 BD adults, 65 SZ adults, and 36 TS adults, we applied hierarchical clustering to the scaling coefficients for the left and right amygdalae, hippocampi, and hemisphere surfaces that significantly differentiated between (1) both BD and SZ adults from HA (*left*), and (2) both TS and SZ adults from HA (*right*). The two largest groups in the left dendrogram were labeled HA (this group consisted of 40 HA) or BD+SZ adults (which consisted of 26 BD adults and 65 SZ adults). The groups in the right dendrogram were labeled HA+TS (which consisted of 40 HA and 35 TS adults) or SZ (which consisted of 65 SZ and 1 TS adults). Because the method performed poorly for these 3-way classifications of adults, we suggested an iterative two-step procedure for classification of these 3 groups. For classifying an individual as a healthy adult, an adult with BD, or an adult with SZ, first an individual was classified as belonging to one of two groups: (1) a healthy adult, or (2) a patient (i.e., as either an adult with BD or an adult with SZ). Our method classified adults between these two groups with 86% sensitivity and 100% specificity. Second, using a different set of scaling coefficients, the individual was classified as either an adult with BD or an adult with SZ with high sensitivity and specificity (**Fig. 2**). Similarly, for classifying an individual as healthy adult, an adults with SZ or an adult with TS, an individual first was classified between two groups: (1) an adult with SZ, and (2) a healthy adult or an adult with TS. Our method classified an adult between these two groups with 99.99% sensitivity and 97.76% specificity. Second, using a different set of scaling coefficients, the individual was classified as either an adult with TS or a healthy adult (**Fig. 3**). *Left* The pattern of the surface features that best classified an individual as either a healthy adult or a patient (i.e. as either an adults with BD or an adult with SZ) in the first 2-way classification included: smaller anterior and dorsal portions of the left amygdala, and smaller anterior and lateral portions of the right amygdala; smaller posterior regions of the left hippocampus, and smaller posterior portions of the right hippocampus; larger dorsolateral prefrontal, smaller ventro-medial, and larger parietal portions of the left hemisphere, and larger superior-parietal, larger superior-occipital, smaller temporal, and smaller medial portions of the right hemisphere. Regions in *red* are local protrusions, and regions in *violet* are local indentations, in BD adults or SZ adults compared with healthy adults. *Right* The pattern of the surface features that best classified an individual into one of the two groups (either as an adult with SZ, or as a healthy adult or adult with TS) included: anterior and lateral regions of the left and right amygdalae; posterior and lateral regions of the left hippocampus, and anterior and posterior regions of the right hippocampus; anterior, ventral, posterior, medial, and superior regions of the left hemisphere, and medial and lateral regions of the right hemisphere. Regions in *red* are local protrusions, and regions in *violet* are local indentations, in SZ adults compared with healthy adults or adults with TS. **LH**, left hemisphere; **RH**, right hemisphere; **AMY**, amygdala; **HC**, hippocampus; **A**, anterior; **P**, posterior.

In contrast to the two-way classifications, the performance of our method for three-way classifications was poor: In discriminating the brains of SZ adults, BD adults, and healthy adults (HA), for example, the adjusted misclassification rates were 0.80, 0.83, and 0.046, for SZ adults, BD adults, and HA, respectively (**Fig. 11** **, left**). However, the brains of SZ adults, BD adults, and HA could be accurately discriminated by applying an iterative, two-way classification strategy in which we first discriminated the brains of HA from those of a combined group of SZ adults and BD adults, and then applying a different set of feature vectors to discriminate the brains of SZ adults from BD adults (**Fig. 8**). Because the brains of SZ adults can be perfectly discriminated from those of BD adults, the misclassification rates for the iterative approach were 0 for HA, 0.14 for BD adult, and 0.14 for SZ adult. Similarly, an iterative, two-way classification strategy was best for discriminating the brains of SZ adults, TS adults, and HA (**Fig. 11** **, right**): we first discriminated the brains of SZ adults from those of a combined group of TS adults and HA, and then used a different set of features to discriminate the brains of TS adults from HA (**Fig. 9**). The misclassification rates for this iterative approach were 0.10 for HA, 0.168 for TS adult, and 0.0245 for SZ adult.

## Discussion

Although previous studies have applied various machine-based classification techniques to brain imaging measures in attempts to diagnose people with various neuropsychiatric illnesses, none to our knowledge have achieved a similar degree of accuracy across as wide a range of neuropsychiatric illnesses as those of the present study. We attribute this success to several unique features of our classification strategy. First, we applied these classification techniques across multiple individually and accurately defined brain regions, rather than to a single image of the entire brain, as is common using techniques such as voxel-based morphometry. Second, we used spherical wavelet transforms to capture spatial patterns of variation in local morphological features, rather than relying on individual and group variability of those local features alone when measured at single isolated voxels. Third, we applied hierarchical clustering techniques to identify natural groupings of spatial patterns of variation in morphological features of the brain across participants, rather than applying those clustering techniques to measures at each individual voxel of the brain. This approach was intended to classify brains according to normal and pathological spatial variations in morphological features that would identify unique, distributed, circuit-based disturbances across the brain associated with specific neuropsychiatric illnesses. [1], [61] The high diagnostic sensitivity and specificity of our classification algorithms across a wide range of disorders and across cohorts of varying sizes, demographic characteristics, and treatment histories, and even in a high risk sample (most of whom were unaffected by manifest illness in their lifetime) demonstrates the exceptional robustness of these methods for imaging-based diagnostic classification of individuals with chronic, well-characterized illness.

Our ability to use only morphological features of the brain to classify and diagnose individuals accurately as having a specific neuropsychiatric illness suggests that the brains of the individuals who share a primary clinical diagnosis also likely share a common core neurobiological substrate for that illness, despite the widely known and undeniable etiologic heterogeneity of virtually all neuropsychiatric disorders. This shared substrate does not mean that the brains of people who have a given neuropsychiatric diagnosis are identical. Indeed, visual inspection of the classification trees shows evidence for variability of feature vectors within diagnostic groupings, and even evidence for the presence of morphological subtypes within clusters of a single clinical diagnosis. That variability could represent either the presence of differing etiologic subtypes within a single diagnostic label or the presence of additional, co-occurring illnesses for persons who share a single primary clinical diagnosis, which is common in clinical samples.

Potential sources of error in classification included errors in the methods for extracting feature vectors from the images. Errors in extracted features would have increased their variance and therefore reduced the statistical power of our algorithms for accurately classifying and diagnosing individual brains. We have previously demonstrated, however, that our methods for spatial normalization of brain regions to the template brain are highly accurate. [31] Similarly, the methods that we used to map surface features conformally onto a unit sphere have also been previously validated. [62] Finally, we computed scaling coefficients using the well-validated wavelet transform. The methods applied in each of the various steps, therefore, have been extensively validated previously, and they computed highly accurate scaling coefficients. Another source of potential error in classification was the overlap of the feature spaces across disorders. We validated the structures discovered in our datasets by using leave-one-out and split-half cross validation procedures, which generally demonstrated very low rates of misclassification in independent datasets and a high level of reproducibility in generating the algorithms used for group classifications.

Although we expected that the accuracy of our classification algorithms would improve significantly by including as many as possible of the brain regions that are components of the many neural circuits distributed across the brain, cost constraints limited our inclusion of only those brain regions that already had been delineated in sufficient number for each disorder at the time when we trained and validated each classification algorithm. We have listed in Table 1 the brain regions initially assessed in the training of each classification algorithm and those that contributed significantly to accurate classification. If and when these algorithms are ever used in future real-world applications, a clinician who had narrowed the diagnostic field for a patient to one of two disorders and who wanted assistance in determining which of those disorders was most likely present would use Table 1 to determine the brain regions that need to be defined on the brain images of that patient and then enter those definitions into the relevant diagnostic algorithm. To determine whether a patient's most likely diagnosis is schizophrenia or bipolar disorder, for example, a clinician would need to obtain precisely defined boundaries of the amygdala, hippocampus, and cerebral hemispheres (regions that contribute to accurate discrimination of these disorders) and then enter those boundary definitions into the classification algorithm for schizophrenia and bipolar disorder.

Because our classification algorithm requires for its input the highly precise delineation of several brain regions, it is yet unsuited for dissemination as a complete and practical tool to aid in clinical diagnosis, for three reasons. First, the manual delineation of brain regions is onerous, requiring 3–4 days of rater time to define the various brain regions used to train and validate each of our classification algorithms. Any potential future application of the classification algorithms in practical clinical settings will require the development either of a more automated tool for region delineation or the availability of a central expert processing center that can define these brain regions with a high degree of precision and at relatively low cost, neither of which is currently available. Nevertheless, with these caveats limiting the real-world clinical implementation and dissemination of these techniques and algorithms, we have provided compelling evidence demonstrating the proof of concept that accurate neuropsychiatric diagnosis in single individuals is possible using anatomical MRIs alone. Given that demonstration, the way forward to real-world clinical implementation and dissemination is clear: we must now develop methods that will make precise region delineations widely available for front-line clinicians. Therefore, along with other investigators, we are designing and testing algorithms that automatically delineate brain regions and extract their image features with sufficiently high precision to maintain the performance of our diagnostic algorithms. Second, the critical assessment, comparison, and benchmarking of various combinations of algorithms for region delineation and diagnostic classification will be important in determining which of these combinations is most accurate and cost-effective for future applications within real-world settings. Third, because our algorithms were tested in chronically ill patients, they will need to be tested in new-onset patients if ever they are to be used in real-world practice, as the greatest diagnostic confusion tends to arise clinically with newly presenting patients. It is possible that the brain features that our algorithms found to be most discriminating among the various diagnostic categories were the consequences of the chronicity of the illnesses or their treatments, in which case they may not be as accurately discriminating in new-onset patients. This possibility notwithstanding, it is worth noting that most psychiatric illnesses are already chronic by the time patients present for initial clinical evaluation [63], [64], with the duration of illness prior to first treatment ranging from 2 years for schizophrenia [65], to 6–8 years for mood disorders and 9–23 years for anxiety disorders [66].

Although our classification algorithms identified patients in specific diagnostic groups with remarkable accuracy, they did so in individuals who had chronic, well-defined illness. We intentionally assessed performance of these algorithms in patients whose illnesses were diagnostically clear and unambiguous because we needed confidence in the accuracy of the ground truth clinical labels with which the results of our automated classification could be compared. Those ground truth clinical labels generally are clearest and least ambiguous in chronic patients, for whom the range of symptoms and their clinical courses have fully evolved over time. [67], [68] The accuracy of those ground truth clinical labels was essential for this initial proof-of-concept demonstration that anatomical images alone can accurately diagnose neuropsychiatric illness. The clinical diagnoses were accurate and unambiguous for the participants with either TS, ADHD, SZ, or BD in our cohort because the participants had chronic, well-characterized psychiatric illnesses that had evolved over an average duration of illness of more than 10 years [69], [70], and their diagnoses had been established using carefully applied, research-based diagnostic instruments (SCID) [44] using DSM-IV-TR criteria. Diagnoses were confirmed by two senior, board-certified clinicians who reached clear consensus for the neuropsychiatric diagnoses [46] for each participant. Moreover, we note that our classification accuracies were greatest, and in fact near perfect, when discriminating between two disorders (i.e., between TS or ADHD, SZ or BD, or SZ or TS), compared with rates when we were discriminating between one patient group and healthy participants (Table 1). We attribute this remarkable accuracy to the fact that when discriminating two patient groups, morphological abnormalities in both groups deviated from normal, so that the vectors representing those abnormalities in feature space for each group were further from one another than were their distances from the vectors in feature space for the healthy participants.

Diagnosing neuropsychiatric illnesses using brain imaging measures alone has the potential to transform the clinical care and research of these conditions. If imaging-based diagnoses prove to be as accurate at the stage of initial diagnosis as they seem to be in the diagnostic classification of our chronically ill patients, they will offer the promise of reducing the cost and morbidity associated with inappropriate treatments that are begun following an incorrect initial clinical diagnosis. In addition, imaging-based classifications will likely facilitate the development of primary or secondary prevention strategies for persons who are at increased risk for developing a neuropsychiatric illness. We have demonstrated the feasibility of identifying people who are at risk for becoming ill by discriminating individuals at high or low risk for familial MDD, a sample that include many individuals who had not yet manifested overt symptoms of illness. One of the most important potential future applications of this work is the use of the natural groupings of brains generated by our algorithms to identify brain-based subtypes within a single clinical diagnosis. Differing neurobiological subtypes of illness likely have differing natural histories and respond differentially to specific therapeutic interventions. Identifying those neurobiological subtypes would thereby facilitate the development of truly individualized plans for clinical care. Finally, brain-based diagnoses and the identification of biological subtypes will reduce the presence of phenocopies that are detrimental to the discovery of the genes that predispose to the development of neuropsychiatric illness.

## Supporting Information

Figure S1 **Distribution of Region Definitions by Raters and Diagnoses.**
(TIF)
