# Supplementary figures and images for: Anatomical Brain Images Alone Can Accurately Diagnose Chronic Neuropsychiatric Illnesses
Source: PLoS One. 2012 Dec 7;7(12):e50698. doi: 10.1371/journal.pone.0050698 (PMC3517530; doi:10.1371/journal.pone.0050698)

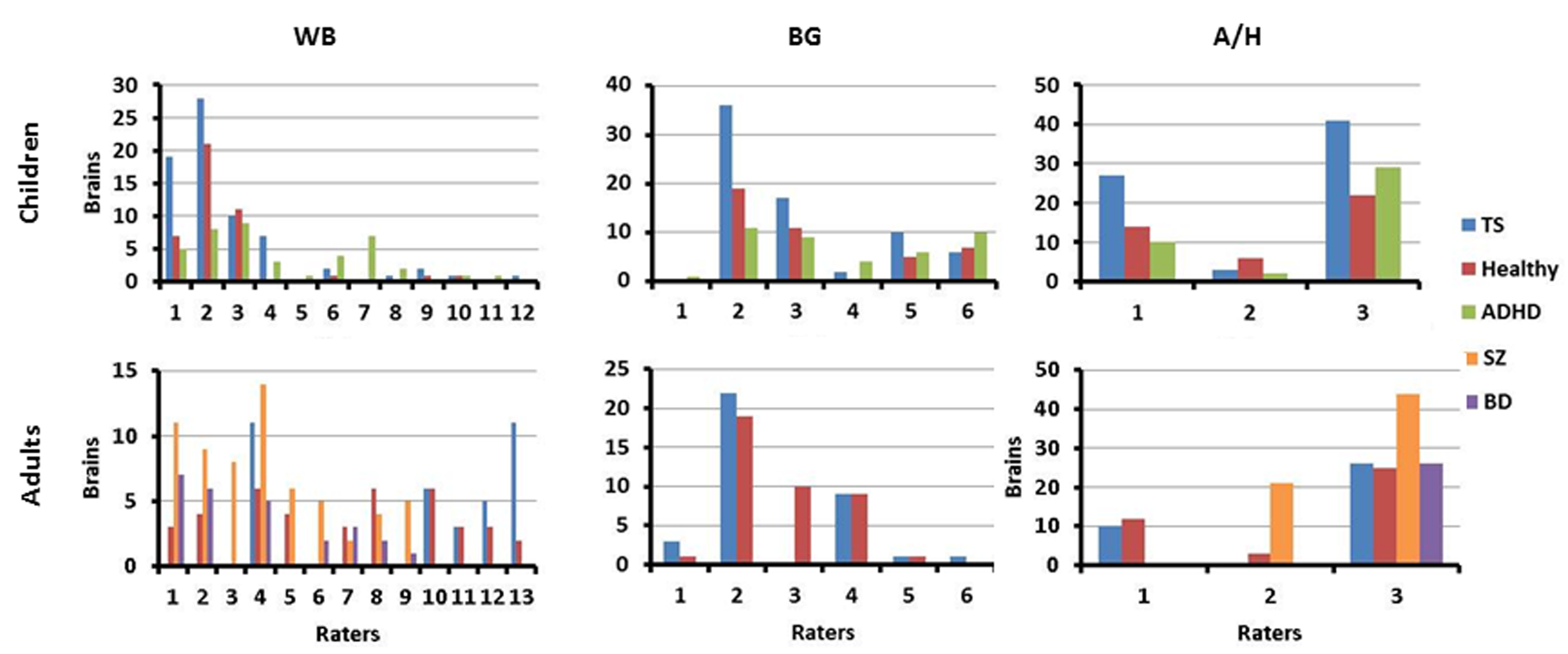

Supplement: Figure S1 — Distribution of Region Definitions by Raters and Diagnoses. (TIF) [file pone.0050698.s001.tif]
